# Effect of Individualized Preventive Care Recommendations vs Usual Care on Patient Interest and Use of Recommendations: A Pilot Randomized Clinical Trial

**DOI:** 10.1001/jamanetworkopen.2021.31455

**Published:** 2021-11-02

**Authors:** Glen B. Taksler, Bo Hu, Frederic DeGrandis, Victor M. Montori, Angela Fagerlin, Zsolt Nagykaldi, Michael B. Rothberg

**Affiliations:** 1 Cleveland Clinic Community Care, Cleveland Clinic, Cleveland, Ohio; 2 Department of Quantitative Health Sciences, Cleveland Clinic, Cleveland, Ohio; 3 Population Health Research Institute, Case Western Reserve University at MetroHealth System, Cleveland, Ohio; 4 Knowledge and Evaluation Research Unit, Mayo Clinic, Rochester, Minnesota; 5 Department of Population Heath Sciences, University of Utah, Salt Lake City; 6 Salt Lake City VA Informatics Decision-Enhancement and Analytic Sciences Center for Innovation, Salt Lake City, Utah; 7 Department of Family and Preventive Medicine, University of Oklahoma, Oklahoma City

## Abstract

**Importance:**

This randomized clinical trial examines the feasibility and acceptability of a decision-making tool for increasing patient interest in individualized recommendations for preventive care services.

**Objective:**

To pilot a tool to help patients compare life expectancy gains from evidence-based preventive services.

**Design, Setting, and Participants:**

This randomized clinical trial examined patient and physician responses to a pilot decision tool incorporating personalized risk factors at 3 US primary care clinics between 2017 and 2020. Eligible patients were between ages 45 to 70 years with 2 or more high-risk factors. Patients were followed-up after 1 year.

**Interventions:**

The gain in life expectancy associated with guideline adherence to each recommended preventive service was estimated. Personalized estimates incorporating risk factors in electronic health records were displayed in a physician-distributed visual aid. During development, physicians discussed individualized results with patients using shared decision-making (SDM). During the trial, patients were randomized to receive individualized recommendations or usual care (nonmasked, parallel, 1:1 ratio).

**Main Outcomes and Measures:**

Primary outcome was patient interest in individualized recommendations, assessed by survey. Secondary outcomes were use of SDM, decisional comfort, readiness to change, and preventive services received within 1 year.

**Results:**

The study enrolled 104 patients (31 development, 39 intervention, 34 control), of whom 101 were included in analysis (mean [SD] age, 56.5 [5.3] years; 73 [72.3%] women; 80 [79.2%] Black patients) and 20 physicians. Intervention patients found the tool helpful and wanted to use it again, rating it a median 9 of 10 (IQR, 8-10) and 10 of 10 (8-10), respectively. Compared with the control group, intervention patients more often correctly identified the service least likely (18 [46%] vs 0; *P* = .03) to improve their life expectancy. A greater number of patients also identified the service most likely to improve their life expectancy (26 [69%] vs 10 [30%]; *P* = .07), although this result was not statistically significant. Intervention patients reported greater mean [SD] improvement in SDM (4.7 [6.9] points) and near-term readiness to change (13.8 points for top-3-ranked recommendations). Point estimates indicated that patients in the intervention group experienced greater, although non–statistically significant, reductions in percentage of body weight (−2.96%; 95% CI, −8.18% to 2.28%), systolic blood pressure (−6.42 mm Hg; 95% CI, −16.12 to 3.27 mm Hg), hemoglobin A_1c_ (−0.68%; 95% CI, −1.82% to 0.45%), 10-year atherosclerotic cardiovascular disease risk score (−1.20%; 95% CI, −3.65% to 1.26%), and low-density lipoprotein cholesterol (−8.46 mg/dL; 95% CI, −26.63 to 9.70 mg/dL) than the control group. Nineteen of 20 physicians wanted to continue using the decision tool in the future.

**Conclusions and Relevance:**

In this clinical trial, an individualized preventive care decision support tool improved patient understanding of primary prevention and demonstrated promise for improved shared decision-making and preventive care utilization.

**Trial Registration:**

ClinicalTrials.gov Identifier: NCT03023813

## Key Points

**Question:**

Do patients benefit from an evidence-based tool individualized for patient risk factors that helps prioritize preventive services based on their potential to improve life expectancy?

**Findings:**

In this pilot randomized clinical trial including 104 patients and 20 physicians, intervention patients found an individualized decision tool helpful and wanted to use it again. Compared with the control group, intervention patients more often correctly identified the service least likely (46.2% vs zero) to improve their life expectancy.

**Meaning:**

An individualized preventive care decision tool was feasible, acceptable, and improved patient understanding of primary prevention.

## Teaser

This randomized clinical trial enrolling US adults examines the feasibility and acceptability of a novel decision tool for aiding adherence to recommended preventive services.

## Introduction

Preventable risk factors contributed to an estimated 61% of US deaths in 2019,^1,2^ and health risks increase with age.^3^ Prior work suggests that optimal preventive care use could add over 2 million healthy life-years nationwide.^4,5^ The US Preventive Services Task Force (USPSTF) recommends 25 preventive services for middle-aged adults aged 50 to 64 years, but in 2015 only 8% of adults 35 years or older received all high-priority services.^6^ A recent study found that adherence to hypertension control, a highly effective intervention, declined over the past decade.^7^

National prevention guidelines present several challenges. First, they scarcely account for individuals’ unique characteristics, such as comorbid conditions, which affect 70% of middle-aged adults.^8^ This standardized approach hinders physicians’ ability to identify which preventive services provide maximum benefit for a specific patient. Second, few guidelines consider patient preferences, including views about side effects, convenience, lifestyle, and cost.^9,10,11^ This generic approach may contribute to low attainment of prevention targets.^8,12^ Third, clinical time is limited, forcing physicians to prioritize among recommended guidelines without having tools to do so. For example, a physician may know that both mammograms and colonoscopies are “life-saving,” but not understand their relative benefits.^13^

 In this pilot study, we evaluated the potential of an individualized decision tool to help patients and physicians better understand the net benefits of all major, USPSTF-recommended preventive services and improve preventive care delivery.

## Methods

We pilot-tested feasibility and interest in a decision tool that was based on a previously published mathematical model that individualizes preventive care recommendations.^14,15^ The model measures change in life expectancy associated with guideline adherence to each of 25 preventive services rated A or B by the USPSTF^9,14,15^ and management of 6 closely related asymptomatic conditions (ie, control of hypertension, hyperlipidemia, diabetes, and overweight or obesity; cessation of tobacco use or alcohol misuse). Results are individualized for patient age, sex, race, medical history, family history, and lifestyle. For example, while studies suggest that in the general population, colorectal cancer screening adds 270 life-years per 1000 individuals (0.27 years per person),^16^ the individualized model assigns greater benefits for patients with family history of colorectal cancer but lower benefits for those with uncontrolled diabetes (because of shorter life expectancy).^17^ Based on patient focus groups and a national survey, we developed a preliminary visual aid to communicate model recommendations.^18^

This study was approved by Cleveland Clinic’s institutional review board (protocol and statistical analysis plan in Supplement 1) and prospectively registered (NCT03023813).^19^ Physician informed consent was obtained. For patients, a waiver of informed consent was issued for use of the decision tool (because physicians retained discretion in ordering) and written or verbal informed consent was obtained before a survey in accordance with institutional review board requirements. We followed Consolidated Standards of Reporting Trials (CONSORT) reporting guideline for trial studies.

### Setting

The study began February 22, 2017, and because of the COVID-19 pandemic, terminated February 17, 2021, at the Cleveland Clinic Health System. (The last participant enrolled March 12, 2020.) The system comprises a large academic medical center, 13 regional hospitals, 21 family health centers, and over 75 outpatient locations. The pilot was conducted at 3 ambulatory clinics: at the main campus—which draws a variety of patient demographics, including many employees—and facilities in underserved East Cleveland (90.3% African American, 41.8% impoverished)^20^ and suburban Beachwood, Ohio.

### Design

The pilot included 2 phases: development and a nonmasked randomized clinical trial (RCT). In the development phase (ended October 31, 2017), we convened a patient-physician advisory panel for 6 meetings (typically 6 to 9 patients and 6 to 7 physicians per session). Topics included general impressions, the tool’s visual design, and shared decision-making.^21,22,23^ We also brainstormed visual design with 8 graphic designers. To account for clinical workflow, we consulted nurses, medical assistants, operational staff, and departmental leadership. Additionally, we enrolled primary care patients to receive individualized preventive care recommendations during regularly scheduled visits. After each encounter, we requested patient and physician feedback to iteratively improve the tool. Finally, after reaching saturation of comments, we transitioned to a pilot RCT comparing individualized preventive care recommendations (intervention) with usual care (control), with an optional postvisit patient survey in both arms.

### Physician Recruitment

During both phases, we recruited physicians through departmental meetings and in-person, telephone, or email requests. Physicians were asked to try the decision tool at least once and spend their usual amount of time discussing preventive care. However, rather than discussing each service sequentially, we asked physicians to utilize the tool to engage in holistic, shared decision-making about the different preventive services available while incorporating patient values and preferences. Study staff reviewed tenets of shared decision-making with each physician^24^ and emailed them a 4-minute video.^25^

### Patients

Eligible patients were between ages 45 and 70 years with 2 or more of the following characteristics or test results: currently smoking, body mass index 25 or above (BMI, calculated as weight in kilograms divided by height in meters squared), blood pressure (BP) 140/90 or above, hemoglobin A_1c_ (HbA_1c_) levels 9% or higher, 10-year atherosclerotic cardiovascular disease risk (ASCVD) risk 7.5% or above, alcohol misuse, depression, history of sexually transmitted infection, and being overdue for colorectal, cervical, breast, or lung cancer screenings. Exclusion criteria were active cancer (other than nonmelanoma skin), end-stage kidney disease, moderate to severe congestive heart failure or chronic obstructive pulmonary disease, and other comorbidities with limited life expectancy.

Approximately once a week, a nurse reviewed appointment schedules for primary care annual wellness visits to identify eligible patients. Wellness visits focused on prevention and lasted 30 to 40 minutes vs 15 to 20 minutes for routine appointments. For each eligible patient, we computed his or her individualized preventive care recommendations and documented them in a research database. Additionally, for each intervention patient, we created a 1-page graphic handout showing the individualized recommendations and provided a copy to the physician.

On the encounter day, a team member approached the patient in a waiting room to introduce the study, inform them that their physician was participating, and invite them to complete an optional postvisit survey in exchange for a $25 gift card. Finally, for intervention patients, the team member placed the individualized recommendations in a bin outside the examination room with hard copies for the physician, patient, and a companion.

### Randomization

A biostatistician (B.H.) generated a block randomization sequence by patient (sizes 2, 4, 6) with a 1:1 parallel allocation ratio. Since we needed to create individualized recommendations in advance, randomization occurred 1 week before scheduled encounters. If a patient did not enroll, that sequence was skipped.

### Outcomes

The primary outcome was patient self-reported interest in individualized preventive care recommendations. Secondary outcomes included use of shared decision-making (using SDM-Q-9^26,27,28^), decisional comfort (decisional conflict scale^29^), readiness to change (transtheoretical model^30^), and preventive services received within 1 year.

Most outcomes were based on survey responses. Patients could complete the survey immediately by computer in a dedicated clinical room, alone or with help from a team member, or by phone or online within 3 days. The survey asked about current health, preventive services discussed during the encounter, preventive services patients thought were the most and least likely to improve their overall health, primary and secondary study outcomes, demographics (ie, age, sex, race [White/Caucasian; Black/African American; Native American, American Indian/Alaska Native; Pacific Islander/Native Hawaiian; and/or Other: free-text], Hispanic ethnicity, marital status, and education), and free-text comments. Self-reported race and ethnicity were included because of lower preventive care utilization in minority populations^31,32,33,34,35,36^; for 12 participants with no response, we used electronic health records (EHR) documentation. Additionally, intervention patients provided impressions of the decision tool in Likert scale and free-text form. Physicians were asked for feedback using their preferred method (in-person, telephone, email, or EHR) on what worked well, what did not work well, and suggestions for improvement. The first and last authors (G.B.T. and M.B.R.) agreed on qualitative themes representative of patient and physician feedback.

To assess preventive care utilization we reviewed patient medical records approximately 1 year postencounter. Data were made available for all encounters within the health system, including visits with specialists. Then, for each preventive service recommended to at least 10 control and 10 intervention patients (except healthy diet and exercise, which had no EHR data), we employed a generalized linear mixed regression model with 1 row per follow-up encounter. The dependent variable was the relevant preventive service outcome (ie, tobacco use, BMI, systolic BP, HbA_1c_, 10-year ASCVD risk, receipt of cancer screenings). Independent variables were receipt of the intervention (yes or no) and baseline value (baseline HbA_1c_), with a random effect for each patient. For binary outcomes, we included a logit link.

### Statistical Analysis

Power was based on use of shared decision-making (SDM-Q-9).^26^ Assuming a baseline mean (standard error) score of 31 (9) on a 45-point scale, 130 surveys provided 80% power to detect a 15% improvement.^26,37^ We employed 2-sided *t* tests (α = .05) but as a pilot study, our main goal was learning whether individualized recommendations showed promise for further development and testing. Secondary outcomes were not adjusted for multiple comparisons and should be considered exploratory. Analyses were conducted in Stata/MP version 15.1 (StataCorp).

## Results

**Figure 1.**
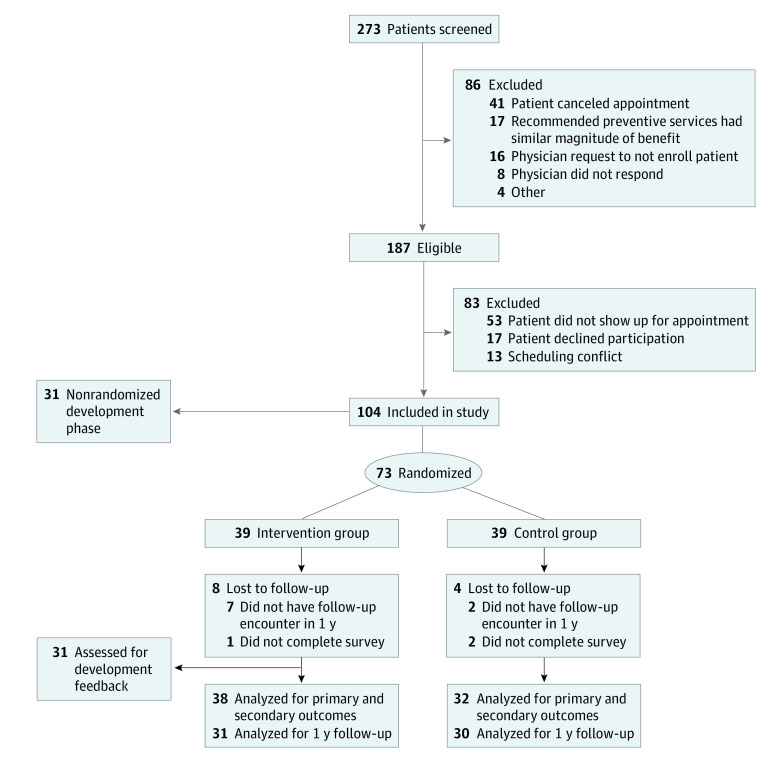
Study Design

We enrolled 104 patients, of whom 31 were in the development phase and 73 were in the RCT (39 intervention, 34 control) (Figure 1). Of these, 101 (97%) completed the survey. Mean (SD) age was 56.3 (5.3) years; most patients were female (73 [72.3%]), Black (80 [79.2%]), and had high school (32 [31.7%]) or some college (29 [28.7%]) education (Table 1). Additionally, we enrolled 20 physicians (of 21 [95%] approached by the study team), including 10 women, 9 from racial or ethnic minority groups, and 8 who immigrated to the US. For the RCT, 61 of 73 (84%) had follow-up visits to assess preventive services received within 1 year. No adverse events were reported to the study team.

**Table 1.** Summary Statistics

Characteristics	Patients, No (%)				
	Overall (N = 101)	Development phase (N = 31)	Randomized clinical trial phase		
			Overall (N = 70)	Control (N = 32)	Intervention (N = 38)
Age, mean (SD), y	56.5 (5.3)	56.9 (4.9)	56.3 (5.6)	56.6 (5.6)	55.9 (5.6)
Practice site					
Main campus (academic medical center, Cleveland, Ohio)	51 (50.5)	14 (45)	37 (53)	17 (53)	20 (53)
Stephanie Tubbs Jones Health Center (underserved East Cleveland, Ohio)	43 (42.6)	17 (55)	26 (37)	12 (38)	14 (37)
Beachwood Family Health Center (suburban Beachwood, Ohio)	7 (6.9)	NA	7 (10)	3 (9)	4 (11)
Sex					
Men	28 (27.7)	8 (26)	20 (29)	7 (22)	13 (34)
Women	73 (72.3)	23 (74)	50 (71)	25 (78)	25 (66)
Race^a^					
White or Caucasian	17 (16.8)	2 (6)	15 (21)	10 (31)	5 (13)
Black or African American	80 (79.2)	28 (90)	52 (74)	22 (69)	30 (79)
Pacific Islander or Native Hawaiian	4 (4.0)	1 (3)	3 (4)	NA	3 (8)
Hispanic					
Not Hispanic	88 (87.1)	30 (97)	58 (83)	26 (81)	32 (84)
Hispanic	2 (2.0)	1 (3)	1 (1)	NA	1 (3)
Missing	11 (10.9)	NA	11 (16)	6 (19)	5 (13)
Education					
Less than high school	6 (5.9)	1 (3)	5 (7)	4 (13)	1 (3)
High school (Diploma or GED)	32 (31.7)	14 (45)	18 (26)	6 (19)	12 (32)
Some college	29 (28.7)	10 (32)	19 (27)	6 (19)	13 (34)
College degree	17 (16.8)	5 (16.1)	12 (17)	9 (28)	3 (8)
Masters degree	8 (7.9)	NA	8 (11)	3 (9)	5 (13)
Doctoral or Professional degree	1 (1.0)	1 (3)	NA	NA	NA
Missing	8 (7.9)	NA	8 (11)	4 (13)	4 (11)
Marital status					
Married or civil/domestic partner	34 (33.7)	8 (26)	26 (37)	12 (38)	14 (37)
Widowed	9 (8.9)	3 (10)	6 (9)	4 (13)	2 (5)
Divorced	19 (18.8)	9 (29)	10 (14)	3 (9)	7 (18)
Separated from spouse or partner	8 (7.9)	2 (6)	6 (9)	3 (9)	3 (8)
Never married or civil/domestic partner	31 (30.7)	9 (29)	22 (31)	10 (31)	12 (32)

Abbreviations: GED, General Educational Development; NA, not applicable.

^a^ Self-reported on patient survey by choosing 1 or more of the following options: White or Caucasian; Black or African American; Native American, American Indian, or Alaska Native; Pacific Islander or Native Hawaiian; Other (please specify). For 12 participants with no response, we used electronic health record documentation. Categories not reported in the table had zero participants.

### Development Phase

**Figure 2.**
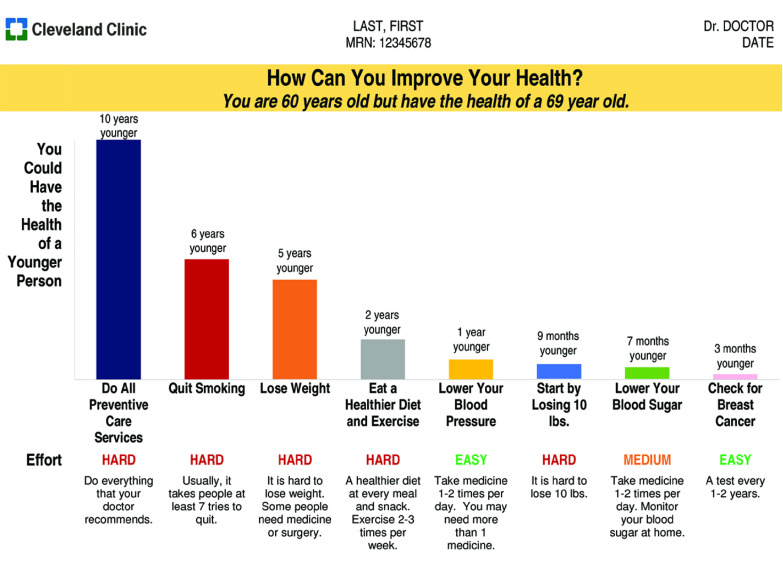
Example of Individualized Preventive Care Recommendations Shown to Patients and Physicians This figure illustrates the final design of the visual aid. Results were individualized for each patient.

In early weeks, patients and physicians found the visual aid too long; we reduced length from 8 pages (eFigure 1 in Supplement 2) to 1 page. After 12 major iterations (eTable 1 in Supplement 2), feedback was consistently positive without further suggestions for change. Figure 2 shows the final design. At top was an individualized statement; eg, “You are 60 years old but have the health of a 69 year old.” To do so, our model estimated a patient’s life expectancy and converted it into “true age,” the age most commonly associated with that life expectancy. Below, a bar graph showed the improvement in true age if a patient obtained all recommended preventive services (eg, “10 years younger”) and the change in true age associated with each service. To avoid overprecision, changes were rounded to the nearest year (or month if less than 1 year), with more than 10 years expressed as, “More than 10 years younger.”

Text below each bar conveyed the effort required to follow the recommendation (easy, medium, or hard) and a short description (eg, “Usually, it takes people at least 7 tries to quit”) based on patient-advisory panel feedback. The final design showed 2 weight loss categories: “Lose Weight” with a 25 BMI goal—a difficult, if not impossible, task for many patients^30^ —and “Start by Losing 10 lbs,” intended to proxy a more achievable 5% weight loss goal.^10,11^ The survey underwent 3 revisions based on patient and physician feedback (eMethods in Supplement 2).

### RCT Phase

Table 2 shows RCT results. Patients were eligible for a median (IQR) 6 (5-6) preventive services. Their true age was a mean (SD) 7.7 (4.0) years older than their biological age. Weight loss, healthy diet and exercise, cholesterol reduction, and colorectal cancer screening were recommended to most patients.

**Table 2.** Results of Randomized Clinical Trial

	Patients, No. (%)			*P* value
	Overall (N = 70)	Control (N = 32)	Intervention (N = 38)	
Age, mean (SD), y				
Age	56.3 (5.6)	56.6 (5.6)	55.9 (5.6)	.63
True age	63.1 (6.9)	64.0 (6.7)	62.3 (7.1)	.36
Difference	7.7 (4.0)	7.4 (4.6)	8.0 (3.5)	.63
No. of preventive services recommended, median (IQR)	5 (5-6)	6 (5-7)	5 (5-6)	.053
Preventive services recommended				
Take cholesterol medicine	58 (83)	21 (66)	17 (45)	NA
Lose weight	56 (80)	26 (81)	30 (79)	
Start by losing 10 lbs	52 (74)	24 (75)	28 (74)	
Eat a healthy diet and exercise^a^	35 (50)	23 (72)	12 (32)	
Check for colon cancer	35 (50)	14 (44)	21 (55)	
Quit smoking	30 (43)	13 (41)	17 (45)	
Take blood pressure medicine	25 (36)	12 (38)	13 (34)	
Check for breast cancer	17 (24)	8 (25)	9 (24)	
Take aspirin^b^	17 (24)	6 (19)	11 (29)	
Lower your blood glucose	15 (21)	5 (16)	10 (26)	
Check for lung cancer	14 (20)	7 (22)	7 (18)	
Check for cervical cancer	10 (14)	7 (22)	3 (8)	
Get a blood glucose test	4 (6)	2 (6)	2 (5)	
Check for osteoporosis	2 (3)	1 (3)	1 (3)	
Decrease your alcohol use	2 (3)	1 (3)	1 (3)	
Get a cholesterol test	1 (1)	0	1 (3)	
Check for abdominal aortic aneurysm	1 (1)	1 (3)	0	
Comprehension of decision support				
Patient correctly chose top-ranked preventive service	37 (52)	10 (30)	26 (69)	.07
Patient correctly chose bottom-ranked preventive service	21 (30)	0	18 (46)	.03
Patient correctly chose magnitude of benefit for top-ranked preventive service	NA	NA	23 (61)	NA
Patient correctly chose magnitude of benefit for bottom-ranked preventive service	NA	NA	15 (39)	NA
Patient correctly stated difference between true age and current age^c^	12 (46)	0	12 (86)	<.001
Interest in individualized preventive care recommendations, median (IQR), 10-point scale				
Overall, how helpful did you find the written material (handouts) (Intervention patients only)	NA	NA	9 (8-10)	NA
In the future, would you like to see updated written materials (handouts) (Intervention patients only)	NA	NA	10 (8-10)	NA
Use of shared decision-making				
SDM-Q-9, mean (SD), 100-point scale	76.9 (28.0)	74.3 (28.2)	79.0 (28.1)	.50
Decisional comfort				
DCS, mean (SD), 100-point scale	56.8 (28.8)	57.8 (29.9)	56.0 (28.3)	.81
Readiness to change scale, mean (SD), 7-point scale				
Top-3 individualized preventive service recommendations				
Over the next 1 mo	6.2 (1.2)	6.0 (1.1)	6.3 (1.4)	.39
Over the next 2-6 mo	6.2 (1.3)	6.1 (1.1)	6.2 (1.5)	.85
Bottom-3 individualized preventive service recommendations				
Over the next 1 mo	5.9 (1.7)	6.2 (1.0)	5.7 (2.1)	.43
Over the next 2-6 mo	6.3 (0.9)	6.2 (0.9)	6.5 (1.0)	.39
Share of preventive services ready to change, mean (SD) score ≥6 on 7-point scale, % of patients				
All individualized preventive service recommendations				
Over the next 1 mo	71.2 (45.7)	69.2 (47.1)	72.7 (45.2)	.77
Over the next 2-6 mo	69.0 (46.7)	68.0 (47.6)	69.7 (46.7)	.89
Top-3 individualized preventive service recommendations				
Over the next 1 mo	78.0 (41.8)	70.8 (46.4)	84.6 (36.8)	.25
Over the next 2-6 mo	78.2 (41.7)	72.7 (45.6)	83.3 (38.1)	.39
Bottom-3 individualized preventive service recommendations				
Over the next 1 mo	73.3 (44.7)	77.8 (42.8)	70.4 (46.5)	.59
Over the next 2-6 mo	75.0 (44.1)	69.2 (48.0)	80.0 (41.4)	.53
Share of preventive services not ready to change, mean (SD) score ≤2 out of 7, % of patients				
All individualized preventive service recommendations				
Over the next 1 mo	5.1 (22.2)	3.8 (19.6)	6.1 (24.2)	.71
Over the next 2-6 mo	15.5 (36.5)	20.0 (40.8)	12.1 (33.1)	.42
Top-3 individualized preventive service recommendations				
Over the next 1 mo	2.0 (14.1)	0	3.8 (19.6)	.34
Over the next 2-6 mo	2.2 (14.7)	0	4.2 (20.4)	.34
Bottom-3 individualized preventive service recommendations				
Over the next 1 mo	8.9 (28.8)	0	14.8 (36.2)	.09
Over the next 2-6 mo	0	0	0	NA
Self-assessment of health^d^				
Excellent	6 (9)	5 (16)	1 (3)	.01
Very good	10 (14)	6 (19)	4 (11)	
Good	23 (33)	6 (19)	17 (45)	
Fair	17 (24)	8 (25)	9 (24)	
Poor	3 (4)	1 (3)	2 (5)	
Missing	11 (16)	6 (19)	5 (13)	

Abbreviations: DCS, Decisional Conflict Scale; NA, not applicable; SDM-Q-9, 9-item Shared Decision-Making Questionnaire.

Details on the rank-order and magnitude of life expectancy gain associated with each individualized preventive service recommendation are shown in eFigures 2 and 3 in Supplement 2, respectively.

^a^ After the randomized trial began, some physicians expressed concern about correlation between diet, exercise, and weight loss, so for some patients we removed this service from the decision tool.

^b^ Beginning in February 2019, we added the following footnote to individualized recommendations for aspirin: “We are less sure about aspirin than other ways to improve your health. New evidence suggests that the benefits of aspirin may be much lower.”

^c^ The following survey question was added late in the study design (26 patients included, 12 control and 14 intervention): “People age [patient’s age] years old have a wide range of health conditions. Some people are in very good health and have the health of a [patient’s age − 10] year old. Other people are in worse health and have the health of a [patient’s age + 10] year old. Based on today’s visit, do you have the health of someone who is: [Choose one:]” with 6 response categories ([Patient’s age − 1] years old or younger, Similar to other [patient’s age] year olds, [patient’s age + 1] to [patient’s age + 2] years old, [patient’s age + 3] to [patient’s age + 5] years old, [patient’s age + 6] to [patient’s age + 9] years old, [patient’s age + 10] years old or older).

^d^ Patients selected a response based on the prompt, “In your opinion, would you say your health is.”

### Comprehension

Intervention patients demonstrated comprehension of the decision tool. Following the visit, compared with controls, intervention patients were more likely to identify the preventive service least likely to improve their life expectancy (18 [46%] vs 0; *P* = .03). A greater number of patients also identified the service most likely to improve their life expectancy (26 [69%] vs 10 [30%]; *P* = .07), although this result was not statistically significant. Additionally, nearly two-thirds (23 [62%]) of intervention patients correctly chose the magnitude of benefit for their top-ranked preventive service (6-category drop-down ranging from “1 month to 6 months younger” to “10 years or more younger”), although only 38.5% correctly chose this magnitude for their bottom-ranked preventive service.

In a question added late in the study, we asked 26 patients to compare their true age with their biological age. Twelve intervention patients (85%) correctly did so compared with no patients in the control group (6-category drop-down ranging from “[age − 1] years old or younger” to “[age + 10] years old or older”).

### Primary Outcome

Intervention patients had strongly favorable impressions of the decision tool. When asked, “Overall, how helpful did you find the written material (handouts)?” and “In the future, would you like to see updated written materials (handouts)?” intervention patients rated the tool a median 9 of 10 (IQR, 8-10) and 10 of 10 (8-10), respectively. In free-text comments (Box), intervention patients expressed high satisfaction with the graphic because it was personalized (eg, “Said I am 62 but [with] the health of a 67 year old, that is 5 years I want back”), well-designed, important (“It showed me the importance of taking medications daily to improve my overall health”), credible and promoted doctor-patient discussion (“It was said and explained to me very well and didn’t make me feel bad”).

Box. **Qualitative Feedback From Patients and Physicians**

**Patients**

**What Did You Like BEST About the Written Material (handouts) and Conversation About It With Your Doctor?**

- **Personalization:** “What I like best about the written material [is] it explains that I am 61 and my health is of a 64 [year old], this is a great concern for me and I plan to work on improving this.”… “Said I am 62 but the health of a 67 year old, that is 5 years I want back”… “Personalized for me as an individual—I really liked that.”
- **Design:** “Very simple to read, don't have to try to figure out”… “It was to the point”… “Easy to understand”… “I think the visual presentation was more impactful than reading a few paragraphs.”… “I like the years for quitting smoking, losing weight etc.”… “Straight to the point.”
- **Importance:** “Well it was a good explanation on how to help me live longer, what I need to do to do that”… “It showed me the importance of taking medications daily to improve my overall health and to get my numbers in safe zone.”… “Teaches you how to become more healthy.”… “It showed me where clearly where I am at in my health.”… “It [helped] me understand better I can add years to my life if I change how I live now.”
- **Credibility:** “True facts given to me.”… “Educating to me, knowing things I have to do to better my situation.”
- **Doctor-Patient Discussion:** “I liked how the directions were explained to me; the doctor asked me questions if I understood.”… “[My doctor] answered all my questions, handout tailored to my medical concerns.”… “It was said and explained to me very well and didn’t make me feel bad.”… “Doctor was engaged and asked me questions about improving my health. I was able to tell her about my action plan and the exercises I was going to do in the near future.”… “She [talked] with you, not at you.”… “We went over each thing that I am dealing with, it was very encouraging.”

**What Did You Like LEAST About the Written Material (handouts) and Conversation About It With Your Doctor?**

- **No concerns:** “Nothing”… “I liked all of it” or similar remarks (54 participants).
- **Design:** [In an early development phase iteration^a^] “It said to lose 58 pounds.”… “I would have liked to [see] more helpful hints like what foods can improve the quality of life.” (2 participants).
- **Other:** [In an early development phase iteration^b^] “Talk to me”… “Need more time to talk”… “The results!!!! I’m a 55 in a 65 [year old’s] body.”

**Physicians**

- **Impactful:** “I like it because it’s so easy to show the patient how they can improve. My patient came back for his 3-month follow-up and he had lost 15 lbs.” Follow-up: “Was that normal for him?” “No, it was because he finally understood how important it was, and how everything fit together. I’d be happy to use it again.”
  “It was great. Patient was agreeable to her preventative studies. I believe the chart helped. Thank you.”
  “The value of this is in clearly communicating the importance of each recommendation to patients.”
- **Compelling:** [Pointing to Screen for Lung Cancer recommendation] “I never would have thought of it, but she met all the criteria … So this reminded me that she should get lung cancer screening.”
  “The value of this is in clearly communicating the importance of each recommendation to patients. It has some information on costs which we don’t often know or think about.”
  “Went very well and patient was very happy. Yes, I would definitely use it. I like the one with information about the years of life gained.”
  [Prior to the publication of new evidence on aspirin^c^] “[A]nd I was like, why isn’t she on aspirin? We had talked about it before but then it slipped through the cracks. It turned out that she'd started it but had some stomach pain. But then this reminded me, and I said to her, why don't you try taking it again every other day, because at least that's something? And she said, ‘Oh, my husband's on aspirin, I'd be willing to try it again.’”
- **Desire for change:** “We have 100 places that we have to look in the chart, but now I can look at this and it’s all on 1 page.”
  “This has to get in Epic.”
  “Overall, I would love if this was expanded to all patients so that we can prioritize our interventions given limited time and resources.”
  “I think it’s a good visual aid. If integrated in Epic, it would be nice if we printed as part of their after visit summary (nice take home after counseling during the visit). It can also be a ‘report card’ type of thing.”
- **Patient-physician discussion:** “Handout [was] useful and we had a nice discussion using it. Patient appreciated the line about effort even though unfortunately wasn’t ready to move further towards stopping smoking.”
  “I think it went well. The patient appreciated it. I liked the individualized breakdown and the graphics to help explain to patient.”

**Support Staff**

- **Impactful:** “The fact that participant could take packet home to read, re-read, and possibly act on was great. Having the practitioner go over packet also allowed them a second opportunity to reinforce teaching and/or touch on an area they may have missed.”
- **Workflow:** [Question: Does the research in any way affect your workflow?] “No, it's perfect. We have it all worked out. We go in, get ‘em set up, the doctor goes in, we go back in to finish up, and then they go into the research room [for the survey].” Follow-up: Is there anything we could do better? “Nope, it's all smooth.”

After each encounter in both the development phase and the randomized clinical trial phase, we asked the patient and physician for feedback. We also periodically asked support staff (eg, nurses, medical assistants, patient registration/check-in) for feedback.

^a^The final version of the decision tool provided 2 weight loss recommendations, “Lose weight” and “Start by losing 10 lbs.”

^b^This feedback was provided in an early version of the decision tool. Physicians stated that too much information was provided, so their focus was on reviewing the items rather than shared decision-making with the patient. This eventually led to a 1-page design that was well received.

^c^After new evidence was released on aspirin and a physician expressed concern, we added a footnote: “[W]e are less sure about aspirin than other ways to improve your health. New research suggests that the benefits of aspirin may be much lower.” After the physician next tested the tool, they responded, “This works well. I don’t think we need to get rid of [the aspirin recommendation] entirely. I have just been a bit less impressed by aspirin lately and my desire to start it in general has gone down.”

### Secondary Outcomes

Patients in the intervention group reported greater use of shared decision-making than those in the control group, although the result was not statistically significant (SDM-Q-9 [SD] score: 79.0 [28.1] vs 74.3 [28.2]; *P* = .50). They also reported plans to prioritize top-ranked services. While more patients in the intervention group expressed readiness to change over the next month compared with controls for the top-3–ranked recommendations, the results were not significant (84.6% [36.8%] vs 70.8% [46.4%]; *P* = .25). Fewer patients in the intervention group indicated readiness for the bottom-3-ranked recommendations, although the result was not statistically significant (mean [SD], 70.4% [46.5%] vs 77.8% [42.8%]; *P* = .29). The latter difference dissipated over a horizon of 2 to 6 months (80.0% [41.4%] vs 69.2% [48.0%]; *P* = .53). Decisional comfort was similar across arms (56.0% [28.3%] vs 57.8% [29.9%]; *P* = .81).

In non-preregistered exploratory analysis, patient perceptions of their own health (the last survey question) significantly differed across arms. Fewer intervention patients rated their health as excellent or very good compared with controls (5 [13%] vs 11 [34%]; *P* = .01).

For preventive service utilization, EHR data were available for 6 services recommended to at least 10 control and 10 intervention patients (eTable 2 in Supplement 2). All coefficients were in the expected direction but, consistent with the study’s pilot nature, none were significant. Intervention patients had greater percentage of body weight lost (−2.96%; 95% CI, −8.18% to 2.28%), systolic BP (−6.42 mm Hg; 95% CI, −16.12 to 3.27 mm Hg), HbA_1c_ (−0.68%; 95% CI, −1.82% to 0.45%), 10-year ASCVD risk (−1.20%; 95% CI, −3.65% to 1.26%) and LDL cholesterol (−8.46 mg/dL; 95% CI, −26.63 to 9.70 mg/dL [to convert to millimoles per liter, multiply by 0.0259]) than controls. Results were not significant for tobacco cessation and colorectal cancer screening.

### Physician Feedback

Physicians found the intervention impactful, compelling, desirable, and helpful (Box). Nineteen of 20 physicians wanted to continue using the decision tool in the future. Physicians remarked that the intervention added about 10 minutes to the encounter for the first few uses, and minimal time thereafter.

## Discussion

We pilot-tested an individualized decision tool to inform patients about the change in life expectancy achievable through adherence to evidence-based preventive services. With support from a patient-physician advisory panel, we designed a tool that was feasible and highly acceptable to primary care patients and physicians. Intervention patients demonstrated greater comprehension of the preventive services most and least likely to improve their life expectancy, and had nonstatistically significant increases in use of shared decision-making, readiness to change, and preventive service utilization as compared with controls.

A wide range of decision aids seek to communicate risk and improve shared decision-making based on patient values and preferences.^38^ These can incorporate individual risk factors but typically focus on single decisions, such as whether to screen for prostate cancer^39^ or take statins.^40,41^ In contrast, our approach addressed all evidence-based preventive services at once. This is the second RCT to demonstrate the promise of our individualized framework. A 2021 study^42^ utilized the same model (but with earlier visual presentation) to enroll patients in a year-long health improvement program led by a single nurse practitioner and health coach. It found that intervention patients utilized 0.53 (95% CI, 0.19-0.86) more preventive services than controls, an outcome consistent with earlier work on health risk assessments and an online prevention portal.^43,44^ The current study further developed our decision tool, established strong patient and physician support, and adapted the tool to clinical workflow with 20 physicians across 3 sites.

Patients had a mean true age 7.7 years older than their biological age, providing important health perspective, and were eligible for 5 to 6 preventive services. Our greatest enrollment barriers were patient cancellations and no shows, comprising 1 in 3 screened patients (consistent with general practice at our 3 sites), but among remaining encounters, only 17 patients and 1 physician declined participation. Postenrollment, intervention patients expressed strong satisfaction with the decision tool and demonstrated greater comprehension of prevention priorities than controls. Similarly, 19 of 20 physicians wanted to keep using the tool and supported its integration into the EHR. Their feedback that the tool added minimal time to the encounter after the first few uses was consistent with earlier studies finding that shared decision-making adds only 2 to 3 minutes to visits.^38^

Finally, early evidence suggests that the decision tool may improve outcomes. Use of shared decision-making exceeded the range found in a meta-analysis of shared decision-making interventions (range, 42-75 on a 100-point scale),^28^ and the tool improved readiness to change for all preventive services. All coefficients were in the expected direction and magnitudes were clinically meaningful but would require further testing to establish efficacy. One possible reason is that our holistic approach affected patients’ understanding of their own health, a hypothesis consistent with intervention patients rating their overall health lower than controls. By opening discussion to all preventive services simultaneously rather than sequentially, we allowed patients to express their goals and constraints (eg, transportation, costs) for health improvement. Another possibility is that the tool helped physicians better understand the relative benefits of preventive services. Prior work suggests that physicians care about the potential of preventive services to improve patient length and quality of life, but they need help individualizing these metrics for specific patients.^13^

Taken together, our findings suggest strong potential for individualized, prioritized recommendations to improve preventive care delivery among middle-aged adults. Future work should seek to confirm our results in a larger RCT and establish whether heightened, individualized understanding of preventive care is sufficient to change behavior over time. This work may be particularly important amid the COVID-19 pandemic, as early evidence suggests increased alcohol misuse and less healthy diet and physical activity.^45,46,47^

### Limitations

This study had several limitations. Pilot sample size was small, and few results were statistically significant. Second, because randomization was not stratified by site, intervention patients had less education than controls. However, because few controls demonstrated comprehension of their preventive care priorities or true age, the difference in education should not have been a meaningful factor. Third, we considered length rather than quality of life, likely understating the importance of glycemic control. Fourth, we tested a paper (rather than EHR-based) version of the tool, which is impractical for large-scale clinical integration.

## Conclusions

In a pilot clinical trial, an individualized preventive care decision-support tool improved patient understanding of primary prevention and demonstrated promise for improved shared decision-making and preventive care utilization. Further testing is needed.

## Notes

Supplement 1.
Trial Protocol

Supplement 2. **eTable 1.** Summary of Major Changes to Decision Tool Design **eTable 2.** Preventive Services Utilized by Patients Following the Index Encounter **eFigure 1.** Example of an Early Version of the Decision Tool **eFigure 2.** Variation in Rank-Order of Individualized Preventive Care Recommendations Across Patients **eFigure 3.** Variation in Magnitude of Life Expectancy Gain From Preventive Services Across Patients **eMethods.** Study Design and Study Materials

Supplement 3.
Data Sharing Statement
