# Supplementary material for: Effect of Individualized Preventive Care Recommendations vs Usual Care on Patient Interest and Use of Recommendations: A Pilot Randomized Clinical Trial
Source: JAMA Netw Open. 2021 Nov 2;4(11):e2131455. doi: 10.1001/jamanetworkopen.2021.31455 (PMC8564576; doi:10.1001/jamanetworkopen.2021.31455)
Supplement: Supplement 1. — Trial Protocol [file jamanetwopen-e2131455-s001.pdf]

Supplement 1. Protocol and Statistical Analysis Plan

Table of Contents

Taksler GB, Hu B, DeGrandis F, Montori VM, Fagerlin A, Nagykaldi Z, Rothberg MB. Effect of Individualized Preventive Care Recommendation vs Usual Care on Patient Interest and Use of Recommendations: A Pilot Randomized Clinical Trial.

Protocol and statistical analysis plan	2
Description of study amendments	7
Amendment 1	8
Amendment 2	12
Amendment 3	15
Amendment 4	19
Amendment 5	23

Protocol and statistical analysis plan

We will conduct pilot testing with physicians and patients at Cleveland Clinic Main Campus and the Stephanie Tubbs Jones (STJ) Community Health Center. ***Patient inclusion criteria:*** Age 40-75 years. We will likely focus on established patients (so that model inputs are more readily available), appointments with a primary care physician and ≥1 risk factor (tobacco use, overweight/obese, hypertension, hyperlipidemia, diabetes, alcohol misuse, depression, history of sexually transmitted infection, or being overdue for colorectal, cervical, breast, or lung cancer screenings), but these criteria will not be requirements. We will prefer appointments for physicals and wellness visits, because of the additional time available for pilot testing, but patients with routine (e.g., 20-minute) appointments will also be eligible. This is necessary because not all patients undergo annual physicals. ***Exclusion criteria:*** Severely limited life expectancy (such as in cancer, CHF, COPD, ESRD).

Decision Aid: Throughout pilot testing, we will iteratively develop and modify a decision aid that encourages shared decision-making around preventive care priorities, between patients and physicians. Figure 1 shows an initial example of a decision aid, which would be customized for a patient personalized for his/her individual risk factors. Figure 1 shows additional examples, and Figure 2 shows example written text that may accompany the decision aid, based on success in prior literature.[21,23,26,48] Throughout pilot testing, we will iteratively revise the decision aid based on its previous success or failure, until we reach a saturation point (when patients and physicians have no further major suggestions for improvement, or when results are consistent within the context of pilot testing).

Figure 1. Examples of Alternate Presentation Metrics
We also will consider Figure 1 without numerical estimates, showing "More" or "Less" Important on the y-axis

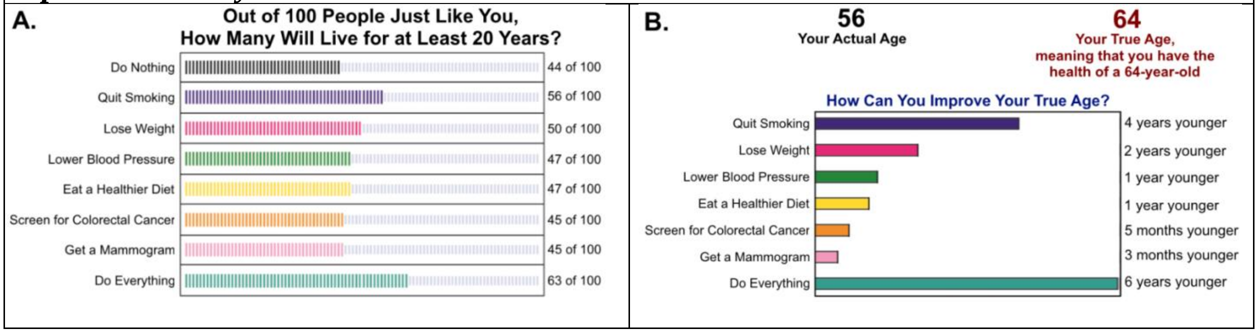

Figure 2. Sample Text Accompanying Graphic in the Decision Aid

	**Ways to Do This**	**Benefits**	**Risks**
Quit smoking	Smoking cessation programs, medicines (such as nicotine gum and patches)	Quitting smoking would lower your risk of a heart attack, heart disease, stroke, and various cancers. You may also cough less and have fewer sore throats.	It is hard to quit smoking. Most people try to quit smoking 7 times before they succeed.

Lower your blood pressure	Medicine, dietary changes (such as eating less sodium or salt), and exercise	You will lower your risk of a heart attack, heart disease, stroke, and kidney disease.	You may have to take medicine every day for the rest of your life.

Training: The study team will lead physician training on shared decision making and use of visual aids, focusing on 3 key steps: informing patient that there are various options for their preventive care, providing more detail about options, and discussing patient preference to decide on a course of action.[8-10] We also will discuss ways to probe patient values in the exam room (e.g., "Are you willing to change your lifestyle to prevent a serious health problem in the future? What kinds of things would you consider? How do you feel about blood tests and X-rays that may be less impactful on your health, but are easier to do?").

Pilot Testing Methods: As part of another IRB-approved study (14-673), an external consultant is developing the PI's mathematical model for personalized preventive care recommendations. For this study, we will use a web-based interface (located on the Cleveland Clinic intranet—no external access), in which a user may input all model parameters and receive the tailored decision aid. Following methods from prior research,[11,12] we then will conduct practice runs (to simulate workflow) and iterative rounds of pilot testing (likely 3 to 4) in short bursts of approx. 4 weeks. To minimize risk of attrition, we will include some but not all physicians (probably 2-4) in each round, so that each provider only needs to participate once. (Physicians may choose to continue based on interest.) Approximately once per week, a research nurse will identify eligible patients who have appointments with participating physicians approximately two weeks later (obtained by a data feed from eResearch.) S/he then will review the chart, manually enter model inputs, and distribute a printed copy of the tailored decision aid to the patient's healthcare team (e.g., to each patient's physician, or to a nurse, medical assistant, secretary or other person designated by each physician). Additional copies will be provided on (or near) the day of appointment. Providers will be asked to show each patient his/her individualized recommendations, engage in shared decision making about preventive care goals, and document use of shared decision making in the patient's chart. At the end of the visit, a medical assistant or research team member will hand the patient a printed copy of the decision aid to take home.

Analysis: We will seek feedback from patients who had appointments with participating physicians during the 4 weeks *before* each pilot testing round (a control group), patients who had appointments *during* pilot testing (intervention group), and participating physicians. We will approach patients in the waiting room prior to appointments, or mail them letters in advance (see attached letter), to ask if they would participate in a 20-minute survey at the end of their visit, in exchange for a $20 gift card. (A survey is attached—oral and written (electronic [REDCAP] and/or pen and paper) versions; approval for both forms of administration are requested.) We may also include parking vouchers. By the time of the survey, intervention patients will have received individualized recommendations, while control patients will not (study staff will still compute the individualized recommendations, but will not share results). Thus, if the intervention group expresses more knowledge of which preventive services are likely to promote longevity, we can attribute this to the intervention. We chose this method as a variant of prior work that has cluster randomized clinical practices to early vs. delayed intervention (where controls were patients in the delayed group before implementation),[12,13] recognizing the non-randomized, exploratory nature of our R21. Outcomes measures (see attached surveys) will

include: patient ability to prioritize preventive services (e.g., “Which of the following do you think is most likely to help you live longer? Least likely? each followed by a list of preventive services), trust in the patient’s physician,[14] readiness to change health behaviors,[15-18] use of shared decision making (Shared Decision Making-Q-9[19]—e.g., “My doctor told me there are different options for preventive care”, “My doctor and I selected preventive care options together”), numeracy,[20,21] and graphical literacy.[22-25] *We hypothesize that patients who received individualized, tailored recommendations will better understand which services are more likely to promote longevity, and engage in more shared decision making*. We also will interview physicians (see attached survey).

Sample Size: Based on a review of clinical schedules, we expect that each participating provider will see 4-32 eligible patients per 4-week pilot round. We will target 130 completed patient surveys, providing 80% power to detect a 15% improvement in use of shared decision making. (We assume a baseline SDM-Q-9 mean and standard error of 31 and 9, respectively, on a 45 point scale.[19,26])

Rapid Improvement: Following each pilot testing round, the study team will review feedback and identify next steps. We will spend approx. 4 weeks improving use of the decision aid, the quality of shared decision making (from both patient and provider perspectives),[27] and workflow before implementing the next round. Testing will stop when feedback suggests that the process cannot be reasonably improved. The finished product will represent a tailored decision aid ready for wide-scale testing.

Informed consent: For use of the decision aid, we request a waiver of informed consent. The intervention is minimal risk because physicians can ignore individualized recommendations, and physicians retain discretion in ordering. However, informed consent will be obtained from patients prior to surveys. Additionally, to better understand how the decision aid facilitates shared decision-making around preventive care, and how the decision aid may be improved, for select appointments (estimated at approximately 20-30) we will either request patient consent to videotape appointments, audiotape appointments or shadow patient appointments. Either the PI or Dr. Jamieson will attend shadow appointments. For these patients, informed consent will be obtained prior to the start of the scheduled medical appointment. Informed consent documents are attached.

Data storage

Cleveland Clinic Center for Value-Based Care Research, Department of Internal Medicine, and Department of Quantitative Health Sciences investigators will collect data as required by the studies discussed within the protocol. Data will be stored on site, on a Cleveland Clinic network drive, and access will be controlled in accordance to Cleveland Clinic and HIPAA standards. Protected Health Information (PHI) will not be shared outside of Cleveland Clinic.

References

1. Centers for Disease Control and Prevention, AARP, American Medical Association. Promoting Preventive Services for Adults 50-64: Community and Clinical Partnerships. Atlanta, GA: National Association of Chronic Disease Directors; 2009.

2. USPSTF A and B Recommendations. U.S. Preventive Services Task Force, October 2014. Accessed at http://www.uspreventiveservicestaskforce.org/Page/Name/uspstf-a-and-b-recommendations/, February 24, 2015.

3. LeBlanc E, O'Connor E, Whitlock EP, Patnode C, Kapka T. Screening for and Management of Obesity and Overweight in Adults. Evidence Report No. 89. AHRQ Publication No. 11-05159-EF-1. Rockville, MD: Agency for Healthcare Research and Quality; October 2011.

4. Leblanc ES, O'Connor E, Whitlock EP, Patnode CD, Kapka T. Effectiveness of primary care-relevant treatments for obesity in adults: a systematic evidence review for the U.S. Preventive Services Task Force. Ann Intern Med 2011 Oct 4;155(7):434-47.

5. Multack M. Use of Clinical Preventive Services and Prevalence of Health Risk Factors Among Adults Aged 50-64: National and State-Level Racial/Ethnic, Socioeconomic, and Health Insurance Coverage Status Disparities. Washington, DC: AARP Public Policy Institute; August 2013.

6. Taksler GB, Keshner M, Fagerlin A, Hajizadeh N, Braithwaite RS. Personalized estimates of benefit from preventive care guidelines: a proof of concept. Ann Intern Med 2013 Aug 6;159(3):161-8.

7. Owens DK, Goldhaber-Fiebert JD. Prioritizing guideline-recommended interventions. Ann Intern Med 2013 Aug 6;159(3):223-4.

8. Edwards A, Elwyn G. Inside the black box of shared decision making: distinguishing between the process of involvement and who makes the decision. Health Expect 2006 Dec;9(4):307-20.

9. Elwyn G, Edwards A, Wensing M, Hood K, Atwell C, Grol R. Shared decision making: developing the OPTION scale for measuring patient involvement. Qual Saf Health Care 2003 Apr;12(2):93-9.

10. Elwyn G, Frosch D, Thomson R, Joseph-Williams N, Lloyd A, Kinnersley P, Cording E, Tomson D, Dodd C, Rollnick S, Edwards A, Barry M. Shared decision making: a model for clinical practice. J Gen Intern Med 2012 Oct;27(10):1361-7.

11. Glasgow RE, Kessler RS, Ory MG, Roby D, Gorin SS, Krist A. Conducting rapid, relevant research: lessons learned from the My Own Health Report project. Am J Prev Med 2014 Aug;47(2):212-9.

12. Krist AH, Glenn BA, Glasgow RE, Balasubramanian BA, Chambers DA, Fernandez ME, Heurtin-Roberts S, Kessler R, Ory MG, Phillips SM, Ritzwoller DP, Roby DH, Rodriguez HP, Sabo RT, Sheinfeld Gorin SN, Stange KC, Group MS. Designing a valid randomized pragmatic primary care implementation trial: the my own health report (MOHR) project. Implement Sci 2013 873.

13. Nagykaldi Z, Aspy CB, Chou A, Mold JW. Impact of a Wellness Portal on the delivery of patient-centered preventive care. J Am Board Fam Med 2012 Mar-Apr;25(2):158-67.

14. Sepucha KR, Fagerlin A, Couper MP, Levin CA, Singer E, Zikmund-Fisher BJ. How does feeling informed relate to being informed? The DECISIONS survey. Med Decis Making 2010 Sep-Oct;30(5 Suppl):77S-84S.

15. Dillard AJ, Ferrer RA, Ubel PA, Fagerlin A. Risk perception measures' associations with behavior intentions, affect, and cognition following colon cancer screening messages. Health Psychol 2012 Jan;31(1):106-13.

16. Prochaska JO, DiClemente CC, Norcross JC. In search of how people change. Applications to addictive behaviors. Am Psychol 1992 Sep;47(9):1102-14.

17. Prochaska JO, DiClemente CC. Stages and processes of self-change of smoking: toward an integrative model of change. J Consult Clin Psychol 1983 Jun;51(3):390-5.

18. Phillips SM, Glasgow RE, Bello G, Ory MG, Glenn BA, Sheinfeld-Gorin SN, Sabo RT, Heurtin-Roberts S, Johnson SB, Krist AH, Group MS. Frequency and prioritization of patient health risks from a structured health risk assessment. Ann Fam Med 2014 Nov-Dec;12(6):505-13.

19. Kriston L, Scholl I, Holzel L, Simon D, Loh A, Harter M. The 9-item Shared Decision Making Questionnaire (SDM-Q-9). Development and psychometric properties in a primary care sample. Patient Educ Couns 2010 Jul;80(1):94-9.

20. Fagerlin A, Zikmund-Fisher BJ, Ubel PA, Jankovic A, Derry HA, Smith DM. Measuring numeracy without a math test: development of the Subjective Numeracy Scale. Med Decis Making 2007 Sep-Oct;27(5):672-80.

21. Zikmund-Fisher BJ, Smith DM, Ubel PA, Fagerlin A. Validation of the Subjective Numeracy Scale: effects of low numeracy on comprehension of risk communications and utility elicitations. Med Decis Making 2007 Sep-Oct;27(5):663-71.

22. Tait AR, Zikmund-Fisher BJ, Fagerlin A, Voepel-Lewis T. Effect of various risk/benefit trade-offs on parents' understanding of a pediatric research study. Pediatrics 2010 Jun;125(6):e1475-82.

23. Tait AR, Voepel-Lewis T, Zikmund-Fisher BJ, Fagerlin A. The effect of format on parents' understanding of the risks and benefits of clinical research: a comparison between text, tables, and graphics. J Health Commun 2010 Jul;15(5):487-501.

24. Tait AR, Voepel-Lewis T, Zikmund-Fisher BJ, Fagerlin A. Presenting research risks and benefits to parents: does format matter? Anesth Analg 2010 Sep;111(3):718-23.

25. Hawley ST, Zikmund-Fisher B, Ubel P, Jancovic A, Lucas T, Fagerlin A. The impact of the format of graphical presentation on health-related knowledge and treatment choices. Patient Educ Couns 2008 Dec;73(3):448-55.

26. Tinsel I, Buchholz A, Vach W, Siegel A, Durk T, Buchholz A, Niebling W, Fischer KG. Shared decision-making in antihypertensive therapy: a cluster randomised controlled trial. BMC Fam Pract 2013 14135.

27. Lloyd A, Joseph-Williams N, Edwards A, Rix A, Elwyn G. Patchy 'coherence': using normalization process theory to evaluate a multi-faceted shared decision making implementation program (MAGIC). Implement Sci 2013 8102.

28. Taksler GB, Braithwaite RS. Developing a composite weighted quality metric to reflect the total benefit conferred by a health plan. Am J Manag Care 2015 Mar;21(3):221-7.

29. Applegate M, Taksler GB, Hajizadeh N, Milavsky KI, Ekeleme C, Fagerlin A, Uhler L, Braithwaite RS. Pilot-testing a new program for providing personalized and patient-centered preventive care. Am J Accountable Care 2014 Dec 12;2(4):64-9.

30. Nattinger AB. In the clinic. Breast cancer screening and prevention. Ann Intern Med 2010 Apr 6;152(7):ITC41.

31. Countdown to the October 1, 2015 ICD-10 Compliance Date. Centers for Medicare & Medicaid Services, April 21, 2015. Accessed at http://www.cms.gov/Medicare/Coding/ICD10/index.html, May 6, 2015.

Description of study amendments

Initial submission	6/30/2016	Approved by Institutional Review Board
Amendment 1	2/21/2017	Shortened length of 3 planned patient surveys (pre-encounter, post-encounter, follow-up).
Amendment 2	3/8/2017	Amended from 3 patient surveys to 1 survey shortly after the index encounter. Added physicians as research subjects. Created physician information sheet. Updated patient information sheet to state that patient's doctor was participating in a research study.
Amendment 3	1/31/2018	Added review of patient medical records to examine preventive service utilization post-index encounter.
Amendment 4	3/8/2018	Added electronic (computer) survey option completed by patient alone or with help from a study team member. Added patient educational materials. Updated patient survey.
Amendment 5	9/11/2019	Updated materials for patient qualitative interviews.
Amendment 6	11/30/2020	Closed randomized trial enrollment (no patients enrolled during COVID-19 pandemic for safety reasons).

Amendment 1

We will conduct pilot testing with approximately 10 physicians (see letters of support) at Cleveland Clinic Main Campus in downtown Cleveland, OH and the Stephanie Tubbs Jones (STJ) Community Health Center, located in the underserved community of East Cleveland, OH. (The number of physicians may increase if necessary to achieve desired sample size.) Both facilities serve a wide range of middle-aged patients, with diversity by race/ethnicity and comorbidity (Table 1).

Table 1. Summary Statistics
Patients aged 50-64 y seen in 2015

		Main Campus	*STJ*
N patients		7,550	2,567
N wellness visits		2,456	402
Gender	Female	58%	58%
Race	Black	47%	84%
Smoker	Current	17%	39%
Obese (BMI ≥30.0)		41%	47%
BP	≥140/90	9%	13%
LDL	100-159	40%	36%
	≥160	6%	5%
Diabetes	Diagnosis	29%	40%
	HbA1c>9	4%	7%
Overdue: colorectal cancer screening		37%	46%
Zip code median income <$25,000		31%	61%
Medicaid		5%	44%

Patient inclusion criteria: Age 45-65 years, established patient (so that model inputs are more readily available), appointment for an annual wellness visit with primary care physician, and ≥2 risk factors (tobacco use, overweight/obese, hypertension, hyperlipidemia, diabetes, alcohol misuse, depression, history of sexually transmitted infection, or being overdue for colorectal, cervical, breast, or lung cancer screenings). Although the USPSTF does not recommend annual physicals, annual wellness visits are currently covered by Medicare and all major insurers, and offer a convenient vehicle to pilot test a shared decision making intervention. In our clinic, wellness visits are the most common reason for appointments by middle-aged adults (scheduled >1 day in advance) and last 40 minutes, allowing enough time for pilot testing. Depending on insurance changes, future versions might be administered at routine visits or by non-physicians. ***Exclusion criteria***: Severely limited life expectancy (cancer, CHF, COPD, ESRD). ***Training***: Outside of regular clinic time, study staff will lead physician training on shared decision making and use of visual aids, focusing on 3 key steps: informing patient that there are various options for their preventive care, providing more detail about options, and discussing patient preference to decide on a course of action.[50,67,68] We also will discuss ways to probe patient values in the exam room (e.g., "Are you willing to change your lifestyle to prevent a serious health problem in the future? What kinds of things would you consider? How do you feel about blood tests and X-rays that may be less impactful on your health, but are easier to do?").

Pilot tests: Simultaneous to Aim 1, we will develop a web portal on which a user may input all model parameters and receive the tailored decision aid. Following methods from prior research,[28,97] we then will conduct practice runs (to simulate workflow) and iterative rounds of pilot testing (likely 3 to 4) in short bursts of approx. 2-4 weeks. To minimize risk of attrition, we will include only a subset of the 10 physicians in each round, so that each provider only participates once. (Physicians may choose to continue based on interest.) Each week, a research nurse will identify eligible patients who have appointments with participating physicians 2-4 weeks later (obtained by data feed from our EMR group; our institution routinely uses this process.) Study staff will contact eligible patients by mail, telephone or in waiting rooms (see attachment). Patients will be asked to complete an eligibility questionnaire to confirm that they are eligible (see below). : Study staff then will review the chart, manually enter model inputs,

and distribute a printed copy of the tailored decision aid to each patient's physician. Additional copies will be provided on the day of appointment. Providers will be asked to show each patient his/her individualized recommendations and engage in shared decision making about preventive care goals. At the end of the visit, a medical assistant or study staff will hand the patient a printed copy of the decision aid to take home, along with an after-visit summary that s/he already provides

Eligibility questionnaire: In addition to our above inclusion/exclusion criteria, in order to be eligible, the participant must:
- Complete an eligibility questionnaire (see attachment)
- Select "Yes" for at least 2 items on the question, "In your opinion, which of the following things are important for YOUR health?"
- NOT select 7 (on a 7-point scale) for ALL items in the question, "In your opinion, which of the following things are you likely to do in the next 1 month?" or the following 2 questions.

The eligibility questionnaire is needed to ensure that enrolled patients are aware that they have multiple health issues, and that patients do not have unreasonable expectations about their ability to manage those conditions over the next 6 months (which would be indicated by a 7 on a 7-point scale for all health items).

Feedback: We will seek feedback from intervention patients and participating physicians. Enrolled patients will be asked to participate in a 20-minute survey at the end of their visit, in exchange for $25, and another 15-minute survey 2 to 4 weeks after their visit, in exchange for another $25 (total $50). Based on prior experience, we estimate that 1/3 to 1/2 of patients will agree. We chose this method as a variant of prior work that has cluster randomized clinical practices to early vs. delayed intervention (where controls were patients in the delayed group before implementation),[1,28] recognizing the non-randomized, exploratory nature of our R21. Outcomes measures (Appendix C) will include: patient ability to prioritize preventive services (e.g., "Which of the following do you think is most likely to help you live longer? Least likely? each followed by a list of preventive services), trust in the patient's physician,[13] readiness to change health behaviors,[5,98-100] use of shared decision making (Shared Decision Making-Q-9[69]—e.g., "My doctor told me there are different options for preventive care", "My doctor and I selected preventive care options together"), numeracy,[11,23] and graphical literacy.[14-17]

Figure 6. Sample Questions for Physician Interviews (Draft, Appendix D)

What value did our approach add to the patient encounter?
What was most/least helpful? How could it be improved?
Did the tool encourage shared decision making with patients? How?
Are you inclined to use the tool in practice? Are there any obstacles?
How did it fit with clinical work flow? How might that be improved?
What other information would be helpful?

We hypothesize that patients who received individualized, tailored recommendations will better understand which services are more likely to promote longevity than before receipt of the intervention. We also hypothesize that patients who receive the intervention will report use of shared decision making. We also will interview physicians (Figure 6).

***Sample Size*:** On average, a 1.0 FTE provider sees 8 eligible patients/week; our median FTE is 0.5 (range: 0.2-1.0). So, each of our providers should see 6-32 eligible patients per approx. 4-week pilot, leaving adequate sample size even if the decision aid is not always utilized. Assuming that controls do not systematically differ from intervention patients (the only difference is the week of an appointment), we expect to **minimize potential bias**. In total, we

expect 130 completed patient surveys (16 patients/provider*10 providers*mean of 33%-50% response rate*2 arms), providing 80% power to detect a 15% improvement in use of shared decision making. (We assume a baseline SDM-Q-9 mean and standard error of 31 and 9, respectively, on a 45 point scale.[69,101]) Informed consent will be obtained for patient surveys and shadowing, but use of the decision aid meets criteria for waiver of informed consent. The intervention is minimal risk because physicians can ignore individualized recommendations, and physicians retain discretion in ordering.

Rapid Improvement: Following each burst, the study team will review feedback and identify next steps. We will spend approx. 1-4 weeks improving use of the decision aid, the quality of shared decision making (from both patient and provider perspectives),[102] and workflow before implementing the next round. Testing will stop after approx. 4 rounds or when feedback suggests that the process cannot be further improved in an exploratory study. The finished product will represent a tailored decision aid ready for wide-scale testing.

Videotaping: At some appointments (particularly early in the study), patients will be asked to allow a member of the research team to shadow (observe) or videotape their appointment with their physician. This will allow the researchers to better understand how the decision aid is being discussed during appointments, and variation across patients/physicians. The informed consent document will allow patients to indicate whether they consent to shadowing, videotaping, both, or neither, and patients will be told that they do not have to allow study staff to observe or videotape their appointments.

Cognitive interviews: Selected patients (particularly early in the study) may be asked to participate in cognitive interviews, to help the study team better understand opinions of the individualized preventive care recommendations. Because these interviews are likely to be short (10-15 minutes), the patients who participate in cognitive interviews will not receive additional compensation.

Informed consent: Informed consent will be obtained at the time of the patient's visit with his/her physician. Study staff will approach eligible patients in the waiting room to obtain consent.

Survey mechanics: The surveys, and eligibility questionnaire, will be administered in RedCAP. Patients will be asked for their email address upon expressing interest in the survey, and will be invited to participate in the survey via email with a personalized survey link. **RedCAP requires an email address to send a survey invitation. However, no Personal Health Information (PHI) will be sent by email, only a link to the survey.** The informed consent document will inform participants of our use of email and ask them to write down their email address. Survey responses will be confidential, not anonymous, in order to allow researchers to compare survey responses with individualized recommendations (for example, did patient opinions of which preventive care services were more important agree with our individualized recommendations, after receipt of the intervention) but answers to the survey questions will not be shared with patient's doctors; participants will be informed of confidentiality.

Data storage*:* Cleveland Clinic Center for Value-Based Care Research investigators will collect data as required by the studies discussed within the protocol. Patient data will be stored on site (in a secure REDCap database) and access will be controlled in accordance to Cleveland Clinic

and HIPAA standards. Protected Health Information (PHI) will not be shared outside of Cleveland Clinic.

Amendment 2

We will conduct pilot testing with approximately 10 physicians (see letters of support) at Cleveland Clinic Main Campus in downtown Cleveland, OH and the Stephanie Tubbs Jones (STJ) Community Health Center, located in the underserved community of East Cleveland, OH. (The number of physicians may increase if necessary to achieve desired sample size.) Both facilities serve a wide range of middle-aged patients, with diversity by race/ethnicity and comorbidity (Table 1).

Table 1. Summary Statistics
Patients aged 50-64 y seen in 2015

		Main Campus	*STJ*
N patients		7,550	2,567
N wellness visits		2,456	402
Gender	Female	58%	58%
Race	Black	47%	84%
Smoker	Current	17%	39%
Obese (BMI ≥30.0)		41%	47%
BP	≥140/90	9%	13%
LDL	100-159	40%	36%
	≥160	6%	5%
Diabetes	Diagnosis	29%	40%
	HbA1c>9	4%	7%
Overdue: colorectal cancer screening		37%	46%
Zip code median income <$25,000		31%	61%
Medicaid		5%	44%

Patient inclusion criteria: Age 45-70 years, established patient (so that model inputs are more readily available), appointment with a primary care physician, and ≥2 risk factors (tobacco use, overweight/obese, hypertension, hyperlipidemia, diabetes, alcohol misuse, depression, history of sexually transmitted infection, or being overdue for colorectal, cervical, breast, or lung cancer screenings). ***Exclusion criteria***: Severely limited life expectancy (cancer, CHF, COPD, ESRD). ***Training***: Outside of regular clinic time, study staff will lead physician training on shared decision making and use of visual aids, focusing on 3 key steps: informing patient that there are various options for their preventive care, providing more detail about options, and discussing patient preference to decide on a course of action.[50,67,68] We also will discuss ways to probe patient values in the exam room (e.g., "Are you willing to change your lifestyle to prevent a serious health problem in the future? What kinds of things would you consider? How do you feel about blood tests and X-rays that may be less impactful on your health, but are easier to do?").

Pilot tests: Simultaneous to Aim 1, we will develop a web portal on which a user may input all model parameters and receive the tailored decision aid. Following methods from prior research,[28,97] we then will conduct practice runs (to simulate workflow) and iterative rounds of pilot testing (likely 3 to 4) in short bursts of approx. 2-4 weeks. To minimize risk of attrition, we will include only a subset of the approx. 10 physicians in each round, so that each provider only participates once. (Physicians may choose to continue based on interest.) Each week, a research nurse will identify eligible patients who have appointments with participating physicians approx. 2-4 weeks later (obtained by data feed from our EMR group; our institution routinely uses this process.) Study staff will contact eligible patients by mail, telephone or in waiting rooms (see attachment). : Study staff then will review the chart, manually enter model inputs, and distribute a printed copy of the tailored decision aid to each patient's physician. Additional copies will be provided on the day of appointment. Providers will be the research subjects. Providers will be asked to show each patient his/her individualized recommendations and engage in shared decision making about preventive care goals. At the end of the visit, a medical assistant or study staff will hand the patient a printed copy of the decision aid to take home, along with an after-visit summary that s/he already provides ***Feedback***: We will seek feedback from participating physicians. Feedback may be in whatever form is most convenient for each provider (oral,

written, email, etc.). Additionally, we will inform patients that their doctor is participating in a research study, and ask patients if they would be interested in providing feedback in the form of a 10-15 minute survey. The patient will **not** be required to complete the survey. If the patient chooses to complete the survey, then s/he will receive $25. We do not require any pre-specified number of patients to agree. We chose this method as a variant of prior work that has cluster randomized clinical practices to early vs. delayed intervention (where controls were patients in the delayed group before implementation),[1,28] recognizing the non-randomized, exploratory nature of our study. For providers, our goal from feedback will be to create a tool that is easily understood, that providers are interested in using, and that providers believe improves the patient visit and facilitates shared decision-making. For patients who choose to complete the survey, outcomes measures (Appendix C) will include: use of shared decision making (Shared Decision Making-Q-9[69]—e.g., "My doctor told me there are different options for preventive care", "My doctor and I selected preventive care options together") and plans for preventive care activity over the next 1 month and 6 months.

**Figure 6.** Sample Questions for Physician Interviews (Draft, Appendix D)
What value did our approach add to the patient encounter?
What was most/least helpful? How could it be improved?
Did the tool encourage shared decision making with patients? How?
Are you inclined to use the tool in practice? Are there any obstacles?
How did it fit with clinical work flow? How might that be improved?
What other information would be helpful?

We hypothesize that providers who utilized individualized, tailored recommendations will find that they facilitate discussions of preventive care and shared decision-making. We hypothesize that patients who received individualized, tailored recommendations will change their intentions around preventive care (which services they intend to do in the next 1 month or 6 months). We also hypothesize that patients who receive the intervention will report use of shared decision making. We also will interview physicians (Figure 6).

Sample Size**:** On average, a 1.0 FTE provider sees 8 eligible patients/week; our median FTE is 0.5 (range: 0.2-1.0). So, each of our providers should see 6-32 eligible patients per approx. 4-week pilot, leaving adequate sample size even if the decision aid is not always utilized. Assuming that controls do not systematically differ from intervention patients (the only difference is the week of an appointment), we expect to **minimize potential bias**. We do not require a pre-specified number of surveys to be completed. (We assume a baseline SDM-Q-9 mean and standard error of 31 and 9, respectively, on a 45 point scale.[69,101]) An information sheet will be used for physicians, and an additional information sheet will also be provided to patients stating that their doctor is participating in a research study. For patients who are interested in providing feedback, the information sheet will also describe the survey. The intervention is minimal risk because physicians can ignore individualized recommendations, and physicians retain discretion in ordering.

Rapid Improvement: Following each burst, the study team will review feedback and identify next steps. We will spend approx. 1-4 weeks improving use of the decision aid, the quality of shared decision making (from both patient and provider perspectives),[102] and workflow before implementing the next round. Testing will stop after approx. 4 rounds or when feedback suggests that the process cannot be further improved in an exploratory study. The finished product will represent a tailored decision aid ready for wide-scale testing.

Cognitive interviews: Selected patients (particularly early in the study) may be asked to participate in cognitive interviews, to help the study team better understand opinions of the individualized preventive care recommendations. Because these interviews are likely to be short (10-15 minutes), the patients who participate in cognitive interviews will not receive additional compensation.

Survey mechanics: The surveys will be administered in RedCAP. Patients will be offered the opportunity to complete the survey immediately after their appointment, on a Cleveland Clinic computer. Alternatively, patients who prefer may complete the survey on their own computer, using the internet. In this case, we will provide patients with the web address and a code required by RedCap. This printout will come directly from RedCap, and an example is enclosed. Finally, if patients prefer, we can ask them the survey questions over the phone. Patients will have 3 weekdays after their appointment to complete the survey. Survey responses will be confidential, not anonymous, in order to allow researchers to know which patients completed the survey for mailing of gift cards. Answers to the survey questions will not be shared with patient's doctors; participants will be informed of confidentiality.

Data storage*:* Cleveland Clinic Center for Value-Based Care Research investigators will collect data as required by the studies discussed within the protocol. Patient data will be stored on site (in a secure REDCap database) and access will be controlled in accordance to Cleveland Clinic and HIPAA standards. Protected Health Information (PHI) will not be shared outside of Cleveland Clinic.

Amendment 3

We will conduct pilot testing of the decision aid. Pilot testing will be conducted in multiple phases, an initial phase for basic feedback and a subsequent phase for more advanced pilot testing.

Phase I pilot testing

Table 1. Summary Statistics
Patients aged 50-64 y seen in 2015

		Main Campus	*STJ*
N patients		7,550	2,567
N wellness visits		2,456	402
Gender	Female	58%	58%
Race	Black	47%	84%
Smoker	Current	17%	39%
Obese (BMI ≥30.0)		41%	47%
BP	≥140/90	9%	13%
LDL	100-159	40%	36%
	≥160	6%	5%
Diabetes	Diagnosis	29%	40%
	HbA1c>9	4%	7%
Overdue: colorectal cancer screening		37%	46%
Zip code median income <$25,000		31%	61%
Medicaid		5%	44%

This initial pilot testing will be conducted with approximately 10 physicians (see letters of support) at Cleveland Clinic Main Campus in downtown Cleveland, OH and the Stephanie Tubbs Jones (STJ) Community Health Center, located in the underserved community of East Cleveland, OH. (The number of physicians may increase if necessary to achieve desired sample size.) Both facilities serve a wide range of middle-aged patients, with diversity by race/ethnicity and comorbidity (Table 1). ***Patient inclusion criteria***: Age 45-70 years, established patient (so that model inputs are more readily available), appointment with a primary care physician, and ≥2 risk factors (tobacco use, overweight/obese, hypertension, hyperlipidemia, diabetes, alcohol misuse, depression, history of sexually transmitted infection, or being overdue for colorectal, cervical, breast, or lung cancer screenings). ***Exclusion criteria***: Severely limited life expectancy (cancer, CHF, COPD, ESRD). ***Training***: Outside of regular clinic time, study staff will lead providertraining on shared decision making and use of visual aids, focusing on 3 key steps: informing patient that there are various options for their preventive care, providing more detail about options, and discussing patient preference to decide on a course of action.[50,67,68] Providers will be encouraged, but not required, to participate in training. We also will discuss ways to probe patient values in the exam room (e.g., "Are you willing to change your lifestyle to prevent a serious health problem in the future? What kinds of things would you consider? How do you feel about blood tests and X-rays that may be less impactful on your health, but are easier to do?").

Best practices for decision aids require a values clarification (e.g., helping patients to think about which aspects matter most to them) and explanation that a patient is free to choose nontreatment. (Source: National Quality Forum. National Standards for the Certification of Patient Decision Aids. Final Report. December 15, 2016.) Therefore, we have created written materials (attached) to explain that their provider would like to discuss some things about which there is no "right" answer; introduce shared decision making; explain that both patients and providers play important roles; their doctor wants them to participate; and prompts to ask questions. Based on provider and patient feedback that it is important not to overwhelm with too much information during the appointment, we may provide this information to patients with the mailed informational letter, shortly after check-in for their appointment , and/or shortly after they finish with their provider (before they leave).

Simultaneous to Aim 1, we will develop a web portal on which a user may input all model parameters and receive the tailored decision aid. Following methods from prior research,[28,97] we then will conduct practice runs (to simulate workflow) and iterative rounds of pilot testing (likely 3 to 4) in short bursts of approx. 2-4 weeks. To minimize risk of attrition, we will include only a subset of the approx. 10 providers in each round, so that each provider only participates once. (Providers may choose to continue based on interest.) Each week, the study team will identify eligible patients who have appointments with participating providers approx. 2-4 weeks later (obtained by data feed from our EMR group; our institution routinely uses this process.) Study staff will contact eligible patients by mail, telephone or in waiting rooms (see attachment). Study staff then will review the chart, manually enter model inputs, and distribute a printed copy of the tailored decision aid to each patient's providers. Additional copies will be provided on the day of appointment. Providers will be the research subjects. Providers will be asked to show each patient his/her individualized recommendations and engage in shared decision making about preventive care goals. At the end of the visit, a medical assistant or study staff will hand the patient a printed copy of the decision aid to take home, along with an after-visit summary that s/he already provides ***Feedback***: We will seek feedback from participating providers. Feedback may be in whatever form is most convenient for each provider (oral, written, email, etc.). Additionally, we will inform patients that their doctor is participating in a research study, and ask patients if they would be interested in providing feedback in the form of a 10-15 minute survey. The patient will **not** be required to complete the survey. If the patient chooses to complete the survey, then s/he will receive $25. We do not require any pre-specified number of patients to agree. We chose this method as a variant of prior work that has cluster randomized clinical practices to early vs. delayed intervention (where controls were patients in the delayed group before implementation),[1,28] recognizing the non-randomized, exploratory nature of our study. For providers, our goal from feedback will be to create a tool that is easily understood, that providers are interested in using, and that providers believe improves the patient visit and facilitates shared decision-making. For patients who choose to complete the survey, outcomes measures (Appendix C) will include: use of shared decision making (Shared Decision Making-Q-9[69]—e.g., "My doctor told me there are different options for preventive care", "My doctor and I selected preventive care options together") and plans for preventive care activity over the next 1 month and 6 months.

Phase II pilot testing

As Phase I pilot testing winds down, the research team will conduct a subsequent phase of pilot testing that is broader and includes randomization. The protocol for Phase II pilot testing is the same as for Phase I, with the following exceptions:

1. Instead of just physicians, pilot testing may be conducted with any primary care provider (e.g., physicians, physician assistants, nurse practitioners, registered nurses),

2. Pilot testing may be conducted at any Cleveland Clinic internal medicine, community internal medicine, or family medicine department. There is no targeted number of practice sites or providers.

3. Shortly before each appointment at which the decision aid may be discussed, the visit will be randomized to "intervention" or "control." During intervention appointments,

the individualized recommendations will be made available to providers as per Aim 1. During control appointments, the research team may still generate individualized recommendations but will not make them available to providers. Regardless of whether the appointment was intervention or control, the patient will still be informed that their doctor is participating in a research study, and we will ask patients if they would be interested in providing feedback in the form of a 10-15 minute survey, as per Aim 1. As with Aim 1, the patient will **not** be required to complete the survey.

4. Patients who choose to complete the survey will be notified that after their appointment, we may review their medical record to see which health care services they receive during the next 1 year. Providers will be similarly notified that we may review the medical records of their study patients. The purpose of this review is to see whether provider conversations had an impact on which preventive services were ultimately provided to patients. To ensure that patients are aware of this, we will only review the medical records of patients who chose to complete the survey. This process presents no more than minimal risk of harm to subjects and involves no procedures for which written consent is normally required outside the research context.

5. We will use a different survey from Phase I pilot testing. While Phase I pilot testing is primarily intended for general feedback on the design of individualized preventive care recommendations, Phase II pilot testing includes validated scales for patient feedback (e.g., the Shared Decision Making (SDM)-Q-9 scale and the Decisional Comfort Scale). The Phase II pilot testing survey uses a different version for males and females because some preventive services only apply to one sex (e.g., screen for cervical cancer).

6. Employees who participated in Phase I pilot testing will be eligible for participation in Phase II and will provide an updated information sheet if they are asked to participate. We attach a script for this discussion. Patients who participated in Phase I pilot testing will not be eligible for participation in Phase II.

Information sheets, a patient information letter and scripts for Phase II pilot testing are attached.

Design aspects that apply to all pilot testing

**Figure 6.** Sample Questions for Provider Interviews (Draft, Appendix D)
What value did our approach add to the patient encounter?
What was most/least helpful? How could it be improved?
Did the tool encourage shared decision making with patients? How?
Are you inclined to use the tool in practice? Are there any obstacles?
How did it fit with clinical work flow? How might that be improved?
What other information would be helpful?

We hypothesize that providers who utilized individualized, tailored recommendations will find that they facilitate discussions of preventive care and shared decision-making. We hypothesize that patients who received individualized, tailored recommendations will change their intentions around preventive care (which services

they intend to do in the next 1 month or 6 months). We also hypothesize that patients who receive the intervention will report use of shared decision making. We also will interview providers (Figure 6).

Sample Size**:** On average, a 1.0 FTE provider sees 8 eligible patients/week; our median FTE is 0.5 (range: 0.2-1.0). As pilot testing, we do not require a pre-specified number of surveys to be completed. (We assume a baseline SDM-Q-9 mean and standard error of 31 and 9, respectively, on a 45 point scale.[69,101]) An information sheet will be used for providers, and an additional information sheet will also be provided to patients stating that their doctor is participating in a research study. For patients who are interested in providing feedback, the information sheet will also describe the survey. The intervention is minimal risk because providers can ignore individualized recommendations, and providers retain discretion in ordering.

Rapid Improvement: Following each burst, the study team will review feedback and identify next steps. As needed, we will spend approx. 1-4 weeks improving use of the decision aid, the quality of shared decision making (from both patient and provider perspectives),[102] and workflow before implementing the next round. Testing will stop after approx. 4 rounds or when feedback suggests that the process cannot be further improved in an exploratory study. The finished product will represent a tailored decision aid ready for wide-scale testing.

Cognitive interviews: Selected patients (particularly early in the study) may be asked to participate in cognitive interviews, to help the study team better understand opinions of the individualized preventive care recommendations. Because these interviews are likely to be short (10-15 minutes), the patients who participate in cognitive interviews will not receive additional compensation.

Survey mechanics: The surveys will be administered in RedCAP. Patients will be offered the opportunity to complete the survey immediately after their appointment, on a Cleveland Clinic computer. Alternatively, patients who prefer may complete the survey on their own computer, using the internet. In this case, we will provide patients with the web address and a code required by RedCap. This printout will come directly from RedCap, and an example is enclosed. Finally, if patients prefer, we can ask them the survey questions over the phone. Patients will have 3 business days after their appointment to complete the survey. Survey responses will be confidential, not anonymous, in order to allow researchers to know which patients completed the survey for mailing of gift cards. Answers to the survey questions will not be shared with patient's doctors; participants will be informed of confidentiality.

Data storage*:* Cleveland Clinic Center for Value-Based Care Research investigators will collect data as required by the studies discussed within the protocol. Patient data will be stored on site (in a secure REDCap database) and access will be controlled in accordance to Cleveland Clinic and HIPAA standards. Protected Health Information (PHI) will not be shared outside of Cleveland Clinic.

Amendment 4

We will conduct pilot testing of the decision aid. Pilot testing will be conducted in multiple phases, an initial phase for basic feedback and a subsequent phase for more advanced pilot testing.

Table 1. Summary Statistics
Patients aged 50-64 y seen in 2015

		Main Campus	*STJ*
N patients		7,550	2,567
N wellness visits		2,456	402
Gender	Female	58%	58%
Race	Black	47%	84%
Smoker	Current	17%	39%
Obese (BMI ≥30.0)		41%	47%
BP	≥140/90	9%	13%
LDL	100-159	40%	36%
	≥160	6%	5%
Diabetes	Diagnosis	29%	40%
	HbA1c>9	4%	7%
Overdue: colorectal cancer screening		37%	46%
Zip code median income <$25,000		31%	61%
Medicaid		5%	44%

Phase I pilot testing

This initial pilot testing will be conducted with approximately 10 physicians (see letters of support) at Cleveland Clinic Main Campus in downtown Cleveland, OH and the Stephanie Tubbs Jones (STJ) Community Health Center, located in the underserved community of East Cleveland, OH. (The number of physicians may increase if necessary to achieve desired sample size.) Both facilities serve a wide range of middle-aged patients, with diversity by race/ethnicity and comorbidity (Table 1). ***Patient inclusion criteria***: Age 45-70 years, established patient (so that model inputs are more readily available), appointment with a primary care physician, and ≥2 risk factors (tobacco use, overweight/obese, hypertension, hyperlipidemia, diabetes, alcohol misuse, depression, history of sexually transmitted infection, or being overdue for colorectal, cervical, breast, or lung cancer screenings). ***Exclusion criteria***: Severely limited life expectancy (cancer, CHF, COPD, ESRD). ***Training***: Outside of regular clinic time, study staff will lead providertraining on shared decision making and use of visual aids, focusing on 3 key steps: informing patient that there are various options for their preventive care, providing more detail about options, and discussing patient preference to decide on a course of action.[50,67,68] Providers will be encouraged, but not required, to participate in training. We also will discuss ways to probe patient values in the exam room (e.g., "Are you willing to change your lifestyle to prevent a serious health problem in the future? What kinds of things would you consider? How do you feel about blood tests and X-rays that may be less impactful on your health, but are easier to do?").

Best practices for decision aids require a values clarification (e.g., helping patients to think about which aspects matter most to them) and explanation that a patient is free to choose nontreatment. (Source: National Quality Forum. National Standards for the Certification of Patient Decision Aids. Final Report. December 15, 2016.) Therefore, we have created written materials (attached) to explain that their provider would like to discuss some things about which there is no "right" answer; introduce shared decision making; explain that both patients and providers play important roles; their doctor wants them to participate; and prompts to ask questions. Four possible versions are attached; we will try different versions and evaluate feedback until we find a preferred version. Based on provider and patient feedback that it is important not to overwhelm with too much information during the appointment, we may provide this information to patients with the mailed informational letter, shortly after check-in for their appointment , and/or shortly after they finish with their provider (before they leave). For patients who receive

the values clarification before their appointment, we may provide their written individualized recommendations at that time and/or during their appointment.

Simultaneous to Aim 1, we will develop a web portal on which a user may input all model parameters and receive the tailored decision aid. Following methods from prior research,[28,97] we then will conduct practice runs (to simulate workflow) and iterative rounds of pilot testing (likely 3 to 4) in short bursts of approx. 2-4 weeks. To minimize risk of attrition, we will include only a subset of the approx. 10 providers in each round, so that each provider only participates once. (Providers may choose to continue based on interest.) Each week, the study team will identify eligible patients who have appointments with participating providers approx. 2-4 weeks later (obtained by data feed from our EMR group; our institution routinely uses this process.) Study staff will contact eligible patients by mail, telephone or in waiting rooms (see attachment). Study staff then will review the chart, manually enter model inputs, and distribute a printed copy of the tailored decision aid to each patient's providers. Additional copies will be provided on the day of appointment. Providers will be the research subjects. Providers will be asked to show each patient his/her individualized recommendations and engage in shared decision making about preventive care goals. At the end of the visit, a medical assistant or study staff will hand the patient a printed copy of the decision aid to take home, along with an after-visit summary that s/he already provides ***Feedback***: We will seek feedback from participating providers. Feedback may be in whatever form is most convenient for each provider (oral, written, email, etc.). Additionally, we will inform patients that their doctor is participating in a research study, and ask patients if they would be interested in providing feedback in the form of a 10-15 minute survey. The patient will **not** be required to complete the survey. If the patient chooses to complete the survey, then s/he will receive $25. We do not require any pre-specified number of patients to agree. We chose this method as a variant of prior work that has cluster randomized clinical practices to early vs. delayed intervention (where controls were patients in the delayed group before implementation),[1,28] recognizing the non-randomized, exploratory nature of our study. For providers, our goal from feedback will be to create a tool that is easily understood, that providers are interested in using, and that providers believe improves the patient visit and facilitates shared decision-making. For patients who choose to complete the survey, outcomes measures (Appendix C) will include: use of shared decision making (Shared Decision Making-Q-9[69]—e.g., "My doctor told me there are different options for preventive care", "My doctor and I selected preventive care options together") and plans for preventive care activity over the next 1 month and 6 months.

Phase II pilot testing

As Phase I pilot testing winds down, the research team will conduct a subsequent phase of pilot testing that is broader and includes randomization. The protocol for Phase II pilot testing is the same as for Phase I, with the following exceptions:

1. Instead of just physicians, pilot testing may be conducted with any primary care provider (e.g., physicians, physician assistants, nurse practitioners, registered nurses),

2. Pilot testing may be conducted at any Cleveland Clinic internal medicine, community internal medicine, or family medicine department. There is no targeted number of practice sites or providers.

3. Shortly before each appointment at which the decision aid may be discussed, the visit will be randomized to “intervention” or “control.” During intervention appointments, the individualized recommendations will be made available to providers as per Aim 1. During control appointments, the research team may still generate individualized recommendations but will not make them available to providers. Regardless of whether the appointment was intervention or control, the patient will still be informed that their doctor is participating in a research study, and we will ask patients if they would be interested in providing feedback in the form of a 10-15 minute survey, as per Aim 1. As with Aim 1, the patient will **not** be required to complete the survey.

4. Patients who choose to complete the survey will be notified that after their appointment, we may review their medical record to see which health care services they receive during the next 1 year. Providers will be similarly notified that we may review the medical records of their study patients. The purpose of this review is to see whether provider conversations had an impact on which preventive services were ultimately provided to patients. To ensure that patients are aware of this, we will only review the medical records of patients who chose to complete the survey. This process presents no more than minimal risk of harm to subjects and involves no procedures for which written consent is normally required outside the research context.

5. We will use a different survey from Phase I pilot testing. While Phase I pilot testing is primarily intended for general feedback on the design of individualized preventive care recommendations, Phase II pilot testing includes validated scales for patient feedback (e.g., the Shared Decision Making (SDM)-Q-9 scale and the Decisional Comfort Scale). The Phase II survey may be conducted electronically, in writing, or by telephone. The written Phase II pilot testing survey uses a different version for males and females because some preventive services only apply to one sex (e.g., screen for cervical cancer).

6. Employees who participated in Phase I pilot testing will be eligible for participation in Phase II and will provide an updated information sheet if they are asked to participate. We attach a script for this discussion. Patients who participated in Phase I pilot testing will not be eligible for participation in Phase II.

Information sheets, a patient information letter and scripts for Phase II pilot testing are attached.

Design aspects that apply to all pilot testing

We hypothesize that providers who utilized individualized, tailored recommendations will find that they facilitate discussions of preventive care and shared decision-making. We hypothesize that patients who received individualized, tailored recommendations will change their intentions around preventive care (which services they intend to do in the next 1 month or 6 months). We

also hypothesize that patients who receive the intervention will report use of shared decision making. We also will interview providers (Figure 6).

**Figure 6.** Sample Questions for Provider Interviews (Draft, Appendix D)
What value did our approach add to the patient encounter?
What was most/least helpful? How could it be improved?
Did the tool encourage shared decision making with patients? How?
Are you inclined to use the tool in practice? Are there any obstacles?
How did it fit with clinical work flow? How might that be improved?
What other information would be helpful?

***Sample Size*:** On average, a 1.0 FTE provider sees 8 eligible patients/week; our median FTE is 0.5 (range: 0.2-1.0). As pilot testing, we do not require a pre-specified number of surveys to be completed. (We assume a baseline SDM-Q-9 mean and standard error of 31 and 9, respectively, on a 45 point scale.[69,101]) An information sheet will be used for providers, and an additional information sheet will also be provided to patients stating that their doctor is participating in a research study. For patients who are interested in providing feedback, the information sheet will also describe the survey. The intervention is minimal risk because providers can ignore individualized recommendations, and providers retain discretion in ordering.

Rapid Improvement: Following each burst, the study team will review feedback and identify next steps. As needed, we will spend approx. 1-4 weeks improving use of the decision aid, the quality of shared decision making (from both patient and provider perspectives),[102] and workflow before implementing the next round. Testing will stop after approx. 4 rounds or when feedback suggests that the process cannot be further improved in an exploratory study. The finished product will represent a tailored decision aid ready for wide-scale testing.

Cognitive interviews: Selected patients (particularly early in the study) may be asked to participate in cognitive interviews, to help the study team better understand opinions of the individualized preventive care recommendations. Because these interviews are likely to be short (10-15 minutes), the patients who participate in cognitive interviews will not receive additional compensation.

Survey mechanics: The surveys will be administered in RedCAP. Patients will be offered the opportunity to complete the survey immediately after their appointment, on a Cleveland Clinic computer. Alternatively, patients who prefer may complete the survey on their own computer, using the internet. In this case, we will provide patients with the web address and a code required by RedCap. This printout will come directly from RedCap, and an example is enclosed. Finally, if patients prefer, we can ask them the survey questions over the phone. Patients will have 3 business days after their appointment to complete the survey. Survey responses will be confidential, not anonymous, in order to allow researchers to know which patients completed the survey for mailing of gift cards. Answers to the survey questions will not be shared with patient's doctors; participants will be informed of confidentiality.

Data storage*:* Cleveland Clinic Center for Value-Based Care Research investigators will collect data as required by the studies discussed within the protocol. Patient data will be stored on site (in a secure REDCap database) and access will be controlled in accordance to Cleveland Clinic and HIPAA standards. Protected Health Information (PHI) will not be shared outside of Cleveland Clinic.

Amendment 5

Patient feedback interview guide

Oral feedback

Hello [NAME], this is [NAME] calling from Cleveland Clinic. I am calling about a recent appointment you had with [provider name] Is now a good time?

IF NO: When would be a good time? [SCHEDULE APPOINTMENT]

Great. You may remember seeing a handout called "How Can You Improve Your Health?" about preventive care recommendations that were created just for you. Afterwards, you completed a survey as part of a research study. I would like to ask you feedback about that, so we can help improve the research for other patients and doctors in the future. The interview would last about 15 to 30 minutes, and we could do it over the phone now or another time that would be good for you. If you participate in the interview, we would also mail you another $25 gift card,

Before we get started, I want to say that we take your feedback very seriously. So far, we have changed the "How Can You Improve Your Health?" materials [NUMBER—currently 8 or 9] times based on suggestions from other patients and providers. So we are interested in both what you liked and what you didn't like. If you loved the research study, great, but if you don't remember it or even if you threw it in the garbage, that's fine too. Those are exactly the kind of comments we want to hear. Is that ok with you? Great, thank you. Okay, so to get started:

[ASK ANY OF THE FOLLOWING QUESTIONS, OR RELATED QUESTIONS, THAT MAY PROVIDE USEFUL FEEDBACK.]

Do you remember seeing the "How Can You Improve Your Health?" handout/recommendations?

Overall, how useful did you find the individualized recommendations?

Can you tell me a bit about the conversation (was it different from usual conversations with your doctor? How so? How was it similar?)

What did you think of the recommendations?

Do you remember any of the specific recommendations on the handout? Can you tell me more about what you thought of them? [Study team may remind patients of the recommendations, if needed.]What do you plan to do with it / what did you do with the recommendations after your visit?

Did you follow any of the recommendations? Which ones?

What led you to choose those services instead of others? Was the handout helpful in making that decision? How?

Were there other recommendations that you thought of changing, but didn't? Can you tell me more about that?

Did you believe the recommendations? Can you please tell me more about that? What would have made them more believable?

Did it change your interest at all in preventive care, how so? [Study team may reference particular recommendations from the individualized recommendations, if needed.]
What else would you like to see from individualized recommendations?

How could we make it better?

Do you think it would be helpful for other patients?

Thinking back to your office visit what else was particularly helpful during the visit, how was that more or less useful than the individualized recommendations, etc.

Is there anything else you would like to share with us?

Thank you for your time. We appreciate your continued participation in the research study.

V 8/5/19
