# Supplementary material for: Effect of Individualized Preventive Care Recommendations vs Usual Care on Patient Interest and Use of Recommendations: A Pilot Randomized Clinical Trial
Source: JAMA Netw Open. 2021 Nov 2;4(11):e2131455. doi: 10.1001/jamanetworkopen.2021.31455 (PMC8564576; doi:10.1001/jamanetworkopen.2021.31455)
Supplement: Supplement 2. — eTable 1. Summary of Major Changes to Decision Tool Design eTable 2. Preventive Services Utilized by Patients Following the Index Encounter eFigure 1. Example of an Early Version of the Decision Tool eFigure 2. Variation in Rank-Order of Individualized Preventive Care Recommendations Across Patients eFigure 3. Variation in Magnitude of Life Expectancy Gain From Preventive Services Across Patients eMethods. Study Design and Study Materials [file jamanetwopen-e2131455-s002.pdf]

Supplemental Online Content

Taksler GB, Hu B, DeGrandis F Jr, et al. Effect of individualized preventive care recommendations vs usual care on patient interest and use of recommendations: a pilot randomized clinical trial. *JAMA Netw Open.* 2021;4(11):e2131455. doi:10.1001/jamanetworkopen.2021.31455

eTable 1. Summary of Major Changes to Decision Tool Design

eTable 2. Preventive Services Utilized by Patients Following the Index Encounter

eFigure 1. Example of an Early Version of the Decision Tool

eFigure 2. Variation in Rank-Order of Individualized Preventive Care Recommendations Across Patients

eFigure 3. Variation in Magnitude of Life Expectancy Gain From Preventive Services Across Patients

eMethods. Study Design and Study Materials

This supplemental material has been provided by the authors to give readers additional information about their work.

© 2021 Taksler GB et al. *JAMA Network Open.*

eTable 1. Summary of major changes to decision tool design

This table summarizes major changes to the decision tool design during the development phase of our study. Figure 2 in the main document shows the final design.

[a]Some versions were tested simultaneously; e.g., version 1 for some patients and version 2 for other patients, to compare and contrast feedback.

**Version**[a]	**Description**
1	Patient asked to choose baseball card(s) describing each preventive service (quit smoking, lose weight, etc.). Patient and provider asked to discuss individualized materials describing the impact of each preventive service on their life expectancy and sub-goals described in version 1. Example in eFigure 1 of this Supplement.
2	Patient asked to choose baseball card(s) best representing their preventive service goals (improve your health, quality of life, live longer, effort, costs, side effects). Patient and provider asked to discuss individualized materials describing the impact of each preventive service on that goal(s).
3	One-page design with horizontal or vertical bars showing the change in life expectancy associated with each preventive service.
4	Version 3 without displaying the quantitative magnitude; instead, an arrow labeled “More Urgent” for services with bigger impact on life expectancy and “Less Urgent” for those with smaller impact on life expectancy.
5	Version 3 with true age representation of life expectancy.
6	Text below each bar added to describe effort required for attainment of each preventive service.

© 2021 Taksler GB et al. *JAMA Network Open.*

7	Based on feedback that weight loss goals were insurmountable, we removed the exact magnitude of weight loss needed to reach body mass index 25 kg/m^2 (e.g., “Lose 84 lbs.”), and replaced it with a generic description, “Lose Weight.”
8	Added a more achievable weight loss preventive service, “Start by Losing 10 lbs.” for obese patients.
9	Added “Congratulations!” if patient’s true age was younger than his/her actual age.
10	Based on feedback that patients did not understand synergies between preventive services, we added a “Do All Preventive Care Services” bar.
11	To improve visual display and avoid false precision, we changed the accompanying text for preventive services with a >10 year change in true age to “More than 10 years,” with a bar height of 10 years.
12	For non-diabetic patients with LDL<130 mg/dL and diabetic patients with LDL<100 mg/dL, we added a footnote explaining reasons for a statin recommendation (e.g., “The American College of Cardiology recommends cholesterol medicine because of your African American race, tobacco use and diabetes.”)

© 2021 Taksler GB et al. *JAMA Network Open.*

eTable 2. Preventive services utilized by patients following the index encounter

As a secondary outcome of the randomized controlled trial, for services recommended to at least 10 control and 10 intervention patients, we conducted manual chart review to assess outcomes within 1 year after the index encounter. Sixty-one of 70 patients (87%) had at least 1 follow-up encounter in this time frame (30/32 control patients [94%] and 31/38 intervention patients [82%]). ASCVD: Atherosclerotic cardiovascular disease. HbA1c: Glycated hemoglobin. LDL: Low-density lipoprotein cholesterol. SBP: Systolic blood pressure.

[a] Table 2 reports the number of patients who received each recommendation, by study arm.

[b] We considered the outcome of interest to be percent change in weight, so assessed weight in log terms. Results for weight in lbs. were coefficient -2.96 (95% CI: -8.18, 2.26, P=0.27).

[c] HbA1c <7.0% achieved in 5 encounters (2 patients) in the intervention arm vs. zero encounters (zero patients) in the control arm.

[d] The US Preventive Services Task Force recommends initiating statins in adults aged 40-75 y with a 10-year ASCVD risk ≥10%, ≥1 cardiovascular disease risk factor and no history of cardiovascular disease.{Force, 2016 #2}

[e] Chosen to represent the approximate reduction in LDL cholesterol expected from statin therapy.

[f] Change in smoking status from Current Every Day to either Current Some Days or Quit; or from Current Some Days to Quit, expressed as an odds ratio.

[g] Regression was not significant. Colorectal cancer screening was received by 15/21 (71%) intervention patients and 6/14 (43%) control patients within 1 year after the index encounter.

Sorted by P-value for continuous outcome

			**Continuous outcome**		**Binary outcome**		
**Preventive service**	**Metric**	**N follow-up encounters (patients)**[a]	**Coefficient (95% CI)**	**P**	**Target**	**Odds Ratio (95% CI)**	**P**
Lose weight	% change in weight (lbs.)[b]	201 (54)	-2.96% (-8.18, 2.26)	0.27	Weight loss ≥10 lbs.	1.73 (0.34, 8.75)	0.51

© 2021 Taksler GB et al. *JAMA Network Open.*

Take blood pressure medicine	SBP (mmHg)	81 (25)	-6.42 (-16.12, 3.27)	0.19	SBP <130/80 mmHg	10.25 (1.33, 17.85)	0.025
					SBP <140/90 mmHg	3.10 (0.89, 10.78)	0.075
Lower your blood sugar	HbA1c (% points)	29 (13)	-0.68 (-1.82, 0.45)	0.24	HbA1c <7.0%	[c]	[c]
Take cholesterol medicine	10-year ASCVD risk (% points)	27 (23)	-1.20 (-3.65, 1.26)	0.34	10-year ASCVD risk <10%[d]	0.17 (0.00, 70.69)	0.57
	LDL (mg/dL)	27 (23)	-8.46 (-26.63, 9.70)	0.36	≥30% LDL reduction[e]	1.08 (0.06, 19.31)	0.96
Quit smoking	-	136 (28)	-	-	Reduced smoking intensity[f]	1.20 (0.03, 45.56)	0.92
Check for colon cancer	-	23 (23)	-	-	Receipt of colorectal cancer screening[g]	2.52 (<0.001, >1000)	0.99

© 2021 Taksler GB et al. *JAMA Network Open.*

eFigure1. Example of an early version of the decision tool

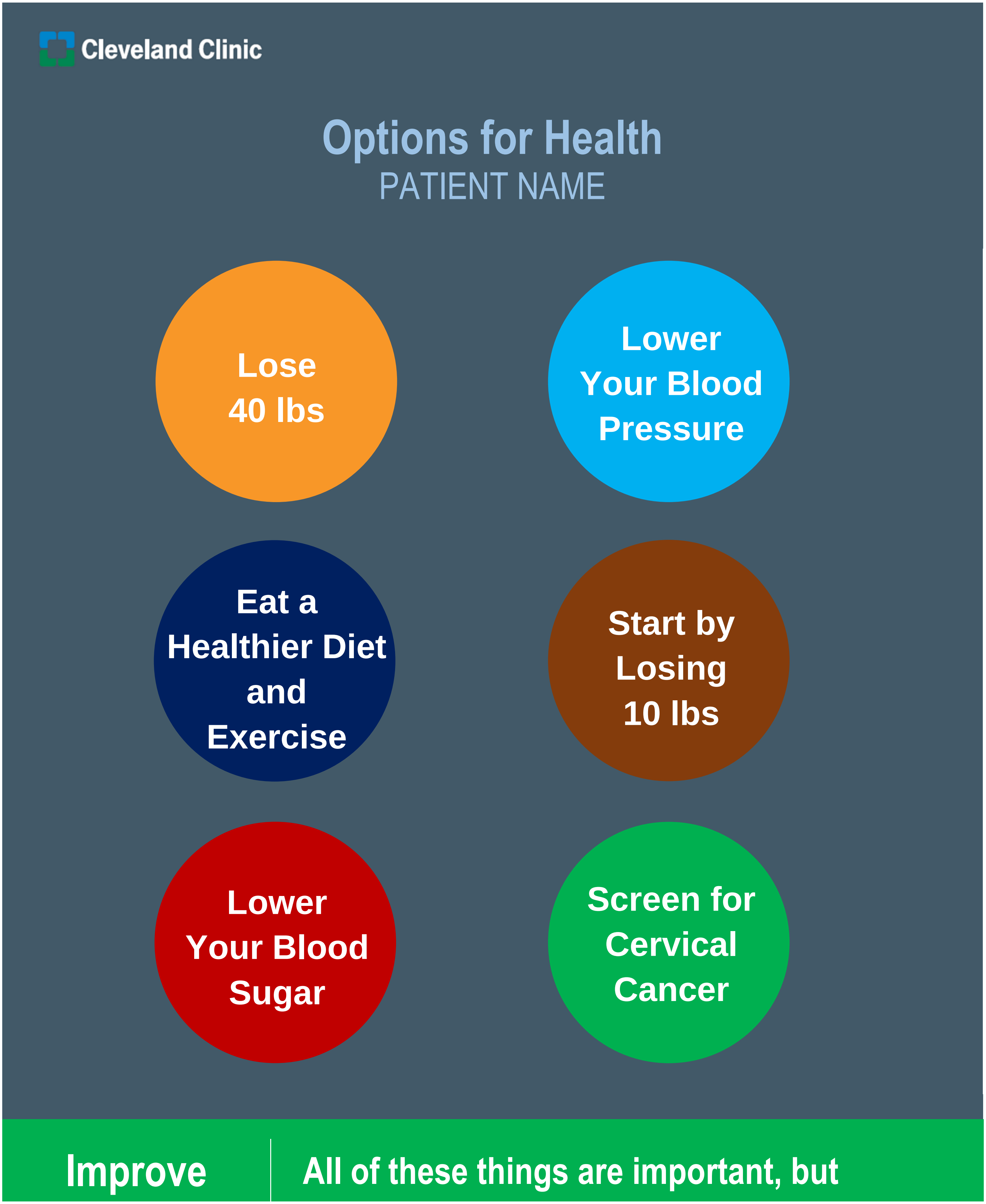

© 2021 Taksler GB et al. *JAMA Network Open.*

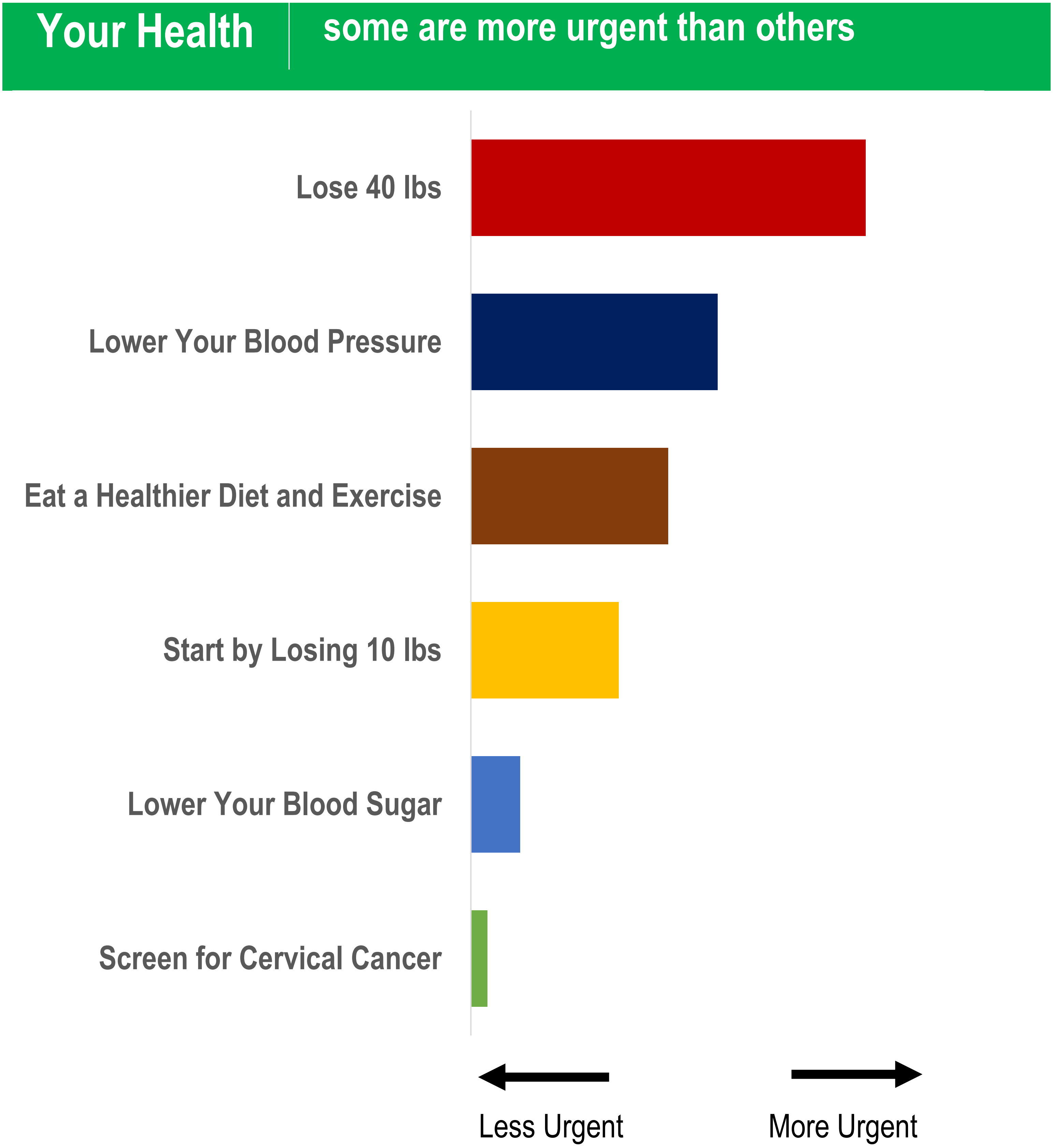

© 2021 Taksler GB et al. *JAMA Network Open.*

Lose 40 lbs

Improve your health

You will have the health of someone **2 years younger**

Quality of life

Help **manage your diabetes,** and **prevent cancer** and **heart disease**. Some people **sleep better** at night.

Live longer

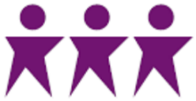

3 lives saved over 10 years, for 100 people *just like you* who lose 40 lbs

Effort

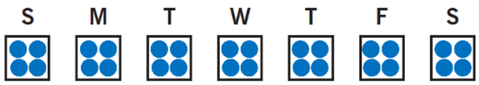

All meals and snacks

Cost

Free programs are available to help you lose weight.
$50-300 per month for popular diet programs.
$150-$250 per month for weight loss medicines.
$15,000-$25,000 for weight loss surgery.

Side effects (Risks)

You may feel **frustrated** and **hungry**.
Weight loss medicines may cause dizziness, high blood pressure and constipation or diarrhea.
Surgery may cause gallstones or bowel obstruction.

Lower Your Blood Pressure

© 2021 Taksler GB et al. *JAMA Network Open.*

Improve your health

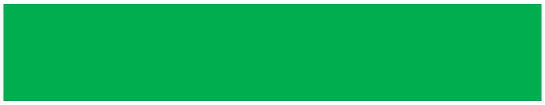

You will have the health of someone **1 year younger**

Quality of life

Help **prevent heart attack** and **stroke**

Live longer

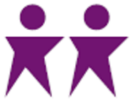

2 lives saved over 10 years, for 100 people *just like you* who lower their blood pressure

Effort

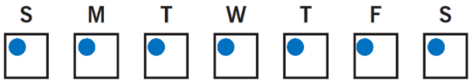

Take medicine 1 time per day

Cost

$4-$25 per month, depending on how many medicines you need.

Side effects (Risks)

More frequent urination, coughing, depression, sleep problems, constipation, or dizziness. If you have a side effect, your doctor **may be able to prescribe a different medicine**.

© 2021 Taksler GB et al. *JAMA Network Open.*

Eat a Healthier Diet and Exercise

Improve your health

You will have the health of someone **1 year younger**

Quality of life

Healthier weight, blood pressure and **blood sugar.** Help **prevent falls, arthritis, heart disease** and some **cancers**.

Live longer

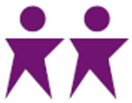

2 lives saved over 10 years, for 100 people *just like you* who eat a healthier diet and exercise

Effort

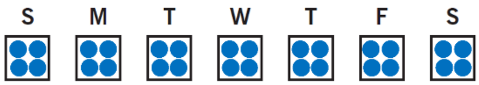

A healthier diet at every meal and snack.
Some examples are **whole grains**, **fruits**, **vegetables**, **healthy proteins** (like poultry, fish and beans) and **low-fat dairy**. Avoid fried foods, saturated fat and sugar.
Exercise 2-3 times per week

Cost

Free programs are available to help you eat more fruits and vegetables.
$0 (free) for exercise you can do yourself, or
$30-$50 per month to join a gym.

Side effects (Risks)

Exercise can cause **muscle injuries, broken bones,** or **heart attacks.** Ask your doctor **how to exercise more safely.**

Start by Losing 10 lbs

© 2021 Taksler GB et al. *JAMA Network Open.*

Improve your health

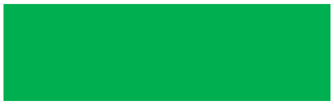

You will have the health of someone **9 months younger**

Quality of life

Help **manage your diabetes,** and **prevent cancer** and **heart disease**. Some people **sleep better** at night.

Live longer

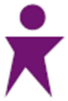

1 life saved over 10 years, for 100 people *just like you* who start by losing 10 lbs

Effort

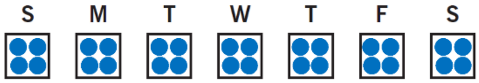

All meals and snacks

Cost

Free programs are available to help you lose weight.
$50-300 per month for popular diet programs.
$150-$250 per month for weight loss medicines.

Side effects (Risks)

You may feel **frustrated** and **hungry**.
Weight loss medicines may cause dizziness, high blood pressure and constipation or diarrhea.

© 2021 Taksler GB et al. *JAMA Network Open.*

Lower Your Blood Sugar

Improve your health

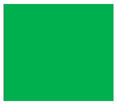

You will have the health of someone **3 months younger**

Quality of life

Help **prevent kidney disease, heart disease, amputations, blindness, numbness** and **tingling**.

Live longer

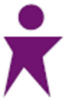

1 life saved over 10 years, for 100 people *just like you* who lower their blood sugar

Effort

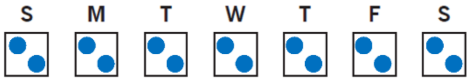

Take medicine 1-2 times per day

Cost

$4-$8 per month for generic medicine

Side effects (Risks)

Sometimes, blood sugar can get too low, causing **shakiness, sweating,** and **rapid heartbeat**.

© 2021 Taksler GB et al. *JAMA Network Open.*

Screen for Cervical Cancer

Improve your health

You will have the health of someone **1 month younger**

Quality of life

Find cancer earlier, when it may be more treatable.

Live longer

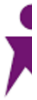

Less than 1 life saved over 10 years, for 100 people *just like you* who screen for cervical cancer

Effort

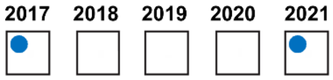

A test every 3-5 years

Cost

Ranges from **$150-$500** depending on your exam

Side effects (Risks)

Over many years, about **25 in 100** women will have a **suspicious finding** that isn't cancer.

© 2021 Taksler GB et al. *JAMA Network Open.*

eFigure 2. Variation in rank-order of individualized preventive care recommendations across patients

This figure shows the rank-order of individualized preventive care recommendations for patients. For example, 1, 2 and 3 indicate the preventive services most likely, second-most likely and third-most likely to improve a patient's life expectancy, respectively.
For each service, the vertical line denotes the median and the rectangle denotes the interquartile range. Whiskers denote 1.5 multiplied by the interquartile range and dots denote outliers.

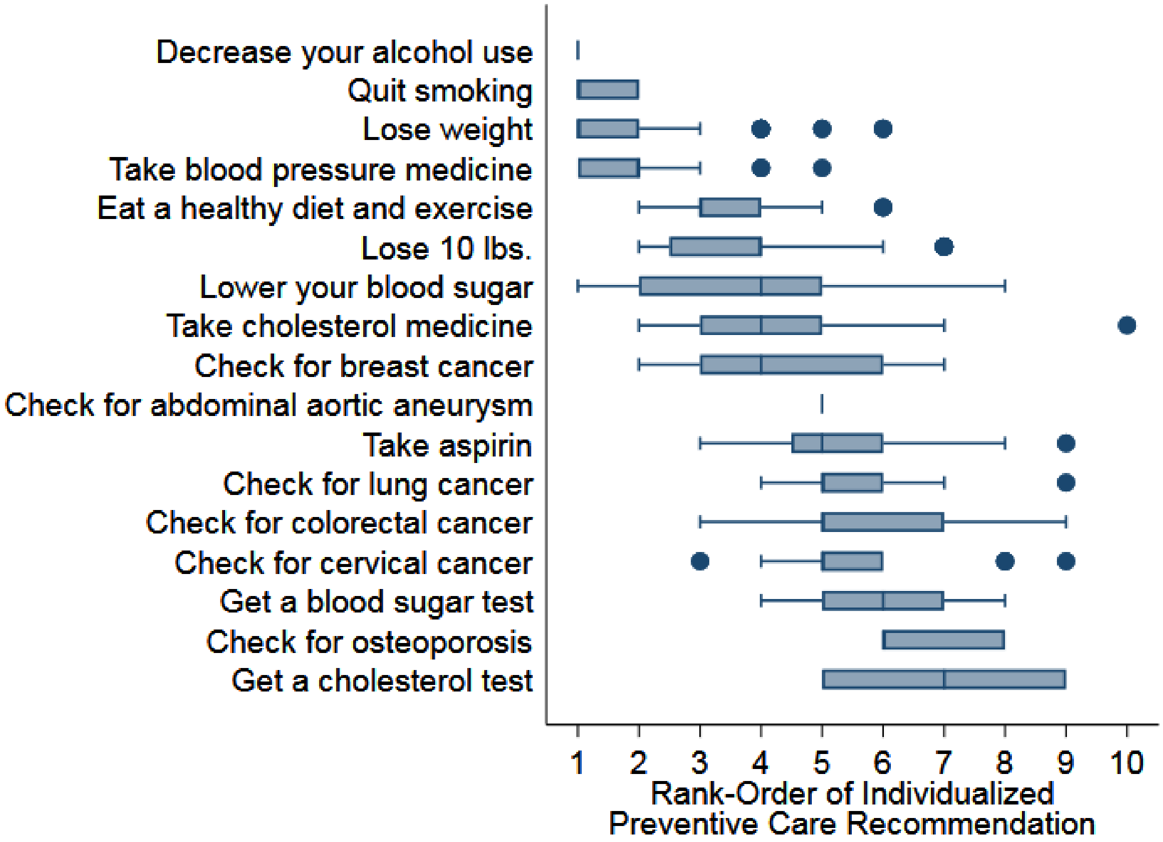

© 2021 Taksler GB et al. *JAMA Network Open.*

eFigure 3. Variation in magnitude of life expectancy gain from preventive services across patients

This figure shows the individualized magnitude of life expectancy gain from preventive services, as estimated by our model, across patients. For example, 1 indicates an estimated life expectancy gain of 1 year, should a patient adhere to that service for the remainder of his/her life, or until recommended cessation by the US Preventive Services Task Force.
For each service, the vertical line denotes the median and the rectangle denotes the interquartile range. Whiskers denote 1.5 multiplied by the interquartile range and dots denote outliers.

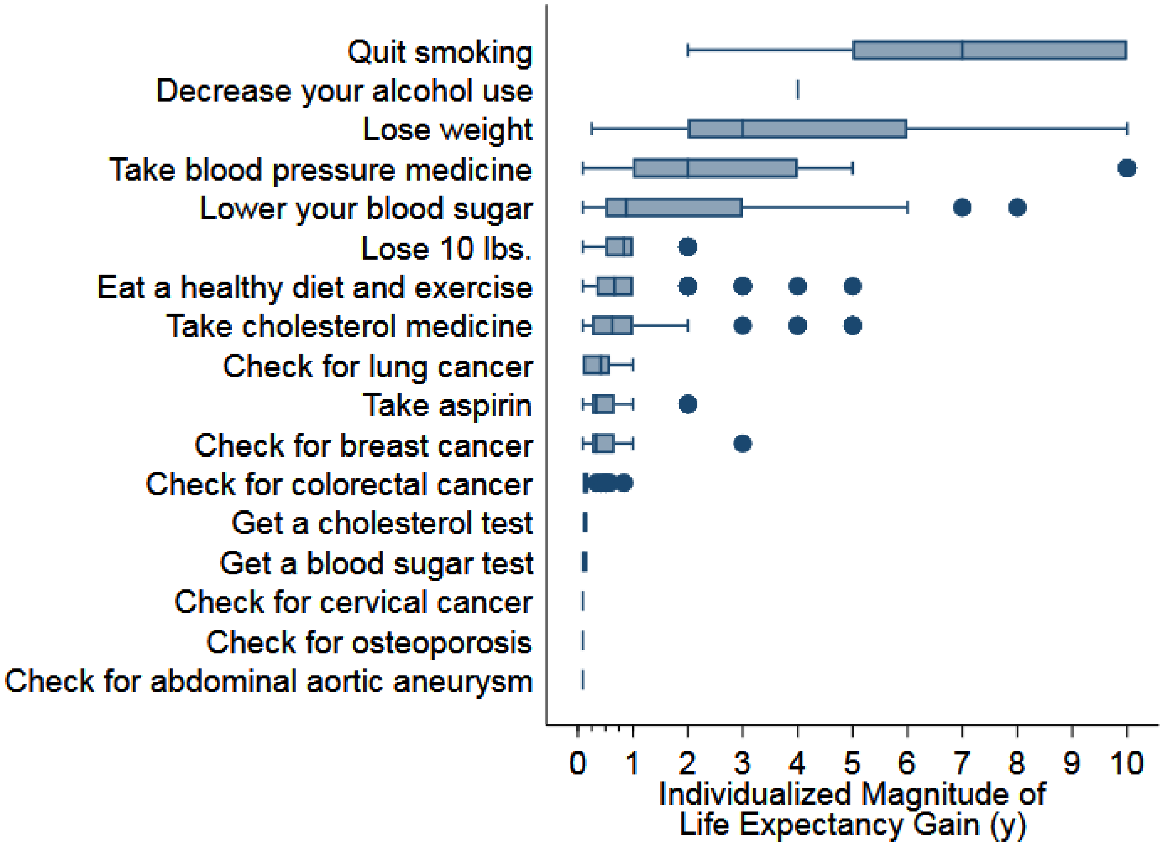

© 2021 Taksler GB et al. *JAMA Network Open.*

eMethods. Study Design and Study Materials

Preregistered study design and outcomes in ClinicalTrials.gov

ClinicalTrials.gov Identifier: NCT03023813

* Study design and current primary and secondary outcome measures were submitted before inception of the randomized trial on March 8, 2018.

Study Design (submitted: February 13, 2018)*

Study Type:	Interventional
Primary Purpose:	Prevention
Study Phase:	Not Applicable
Interventional Study Model:	Parallel Assignment
	Multiple round pilot testing. Some round(s) will have a single group with 1 arm. Other round(s) will be parallel with 2 arms.
Number of Arms:	2
Masking:	None (Open Label)
Allocation:	Randomized
Enrollment:	130 [Anticipated]

Current Primary Outcome Measures (submitted: February 13, 2018)*

Interest in individualized preventive care recommendations. [Time Frame: Within 3 days of index primary care appointment.]

> Measured by individualized preventive service recommendations, tailored to patient's specific medical conditions. Measured by surveys.

Original Primary Outcome Measures (submitted: January 13, 2017)

Understanding of which preventive care services are most likely to promote longevity. [Time Frame: Within one month of primary care visit.]

Current Secondary Outcome Measures (submitted: February 13, 2018)*

Use of shared decision making. [Time Frame: Within 3 days of index primary care appointment.]

> Measured by surveys.

© 2021 Taksler GB et al. *JAMA Network Open.*

Patient readiness to change health behaviors. [Time Frame: Within 3 days of index primary care appointment.]

Measured by surveys.

Decisional comfort. [Time Frame: Within 3 days of index primary care appointment.]

Measured by surveys.

Preventive services received by patients. [Time Frame: Within 1 year of index primary care appointment.]

Measured by chart review.

Original Secondary Outcome Measures (submitted: January 13, 2017)

Use of shared decision making. [Time Frame: On day of primary care appointment where decision aid is presented.]

Patient readiness to change health behaviors. [Time Frame: On day of primary care appointment where decision aid is presented.]

Patient Study Information Sheet

Project Title: Individualizing Disease Prevention for Middle-Aged Adults
Principal Investigator: Glen Taksler, PhD (216-445-7499)

Your health care provider is participating in a research study about different ways to discuss health with patients. At your visit, your provider may show you some written materials and discuss them with you. If so, the written materials were created *just for you* based on your health needs and risk factors. No other patient will receive the same materials.

If you don't see anything new, that's ok. About half of patients will get their usual care.

Please be aware that we are only evaluating **the way** in which your health care provider discusses health with you. The specific recommendations that your health care provider makes are **not** part of the study.

We would like to invite you to participate in the research study as well. If you agree, we will ask you to complete a 10-15 minute survey after your visit. You may answer the questions before you leave, or within 3 days. We will give you a $25 gift card for your time.

If you choose to participate, we will also review your medical record to see what health care services you receive within 1 year after today's visit. This will help us find out whether different ways to discuss health care eventually change patients' health.

If you are interested in participating, this information sheet gives you information about the study. The study staff can review this information with you. The study staff will explain the purpose of the study, any risks to you and what is expected of you. You are free to ask questions about the study at any time. This study has been approved by Cleveland Clinic's Institutional Review Board. Before you learn more about the study, it is important for you to know that your participation in this study is entirely voluntary. You may decide not to take part in, or to withdraw from, the study at any time.

Why are you being asked to participate in this study?
You are being asked to participate because your health care provider is also participating in a research study.

What is the purpose of this study?
The primary objective of this study is to help patients compare the benefits of various preventive care services (things you can do to prevent future health care problems). We are asking health care providers to try discussing the information in different ways, to see what works best.

© 2021 Taksler GB et al. *JAMA Network Open.*

What are the requirements and time commitment to participate?
There are **no requirements** for you. However, if you would like to participate, you will be asked to participate in a survey. The survey will take about 10-15 minutes. You may complete the survey immediately after your visit today or later on your own computer (using the internet). Or, if you prefer, we can ask you the questions over the phone. If you complete the survey on your own computer or by phone, you will need to do so within 3 days.

Also, we will review your medical record to see what health care services you receive within 1 year after today's visit. This will help us find out whether different ways to discuss health care eventually change patients' health.

You may also be asked to participate in an optional interview asking about your opinions on the written materials and discussion with your doctor. We only plan to interview a small number of patients, so please don't be surprised you are not asked to participate in this part of the study. The main purpose of the interview is to help the research team improve the study in the future, for other patients and doctors who may participate. If you agree to an interview, it may take place over the phone or in person, up to 1 year after your appointment with your doctor. The interview will take about 15 to 30 minutes and may be audio recorded.

What are the benefits to taking part in the study?
There is no direct benefit to you. Participating in this study may help researchers gain further knowledge about the best ways to discuss preventive care services with patients.

Are there any risks to you in participating in the study?
There are no physical risks associated with the study. Some of the questions asked as part of the survey or the optional interview may make you feel uncomfortable. You may refuse to answer any of the questions. There is also the possibility that the survey or optional interview questions could cause psychological stress or fatigue to you. You may stop the survey or optional interview at any time.

There is a potential risk of loss of confidentiality of your data. Every effort will be made to keep your information confidential through the use of the following safeguards: All data will be stored in password-protected and encrypted storage facilities accessible only by the research team. Participant names will be replaced by a study number so that individuals who do not need to know your name will not be able to connect your name with the research data.

If you participate in an interview, it may be audio recorded for purposes of transcription (writing) which is needed for research analysis. Your audio recording will not be sent outside of Cleveland Clinic; the transcription (or written copy of the interview) will be made by a member of the Cleveland Clinic research team. Only de-identified (anonymous) text quotes will appear in manuscripts or publications; your audio recording (voice) will not be presented publicly.
The study team may include quotations from your interview in research publications and presentations, but the quotations will not include any information that could identify you.

Do I have to participate in the survey or interview?

© 2021 Taksler GB et al. *JAMA Network Open.*

Your participation is strictly voluntary. Your decision to participate in the survey or interview will not impact your current or future medical care at Cleveland Clinic. You may choose not to take part or may stop the survey or interview at any time. Stopping the survey or interview will not result in any penalty. You may also choose to participate in the survey but not the interview.

What other options are there?
Your participation is strictly voluntary. The alternative is to not participate in the survey.

What are the costs?
There are no costs for you to participate. If you choose to participate, you will receive a $25 gift card after completing the survey.

If you participate in the in-person or phone interview, you will receive an additional $25 gift card, for a total of $50. If you complete the interview by phone, we will mail this gift card to you.

Who do I contact if I have questions about the study?
If you have questions about the study after you have agreed, you may ask the study coordinator Jackie Fox, RN for assistance at any time (216-444-4590) or you can contact Glen Taksler, PhD (principal investigator) at 216-445-7499. If you have any questions about your rights as a research subject, you may contact the Institutional Review Board at (216) 444-2924.

Version 9/11/19

© 2021 Taksler GB et al. *JAMA Network Open.*

Employee Study Information Sheet

Project Title: Individualizing Disease Prevention for Middle-Aged Adults
Principal Investigator: Glen Taksler, PhD (216-445-7499)

Thank you for taking the time to review this information.

This information sheet gives you information about the study which the study staff will then review with you. The study staff will explain the purpose of the study, any risks to you and what is expected of you. You are free to ask questions about the study at any time. This study has been approved by Cleveland Clinic's Institutional Review Board. Before you learn more about the study, it is important for you to know that your participation in this study is entirely voluntary. You may decide not to take part in, or to withdraw from, the study at any time.

Why are you being asked to participate in this study?
You are being asked to participate in this study because you are a health care provider at Cleveland Clinic in adult primary care services.

What is the purpose of this study?
The primary objective of this study is to better understand how health care providers can help adults to make an informed decision about services that are likely to help them live longer, healthier lives, based on their individual needs and risk factors.

What are the requirements and time commitment to participate?
We will randomize select patient appointments to "intervention" or "usual care". For intervention appointments, we will provide you with written materials that describe various preventive care services for patients. The written materials are *different for each patient* based on their disease risk factors. We ask you to discuss the written materials with patients and engage in shared-decision making. For usual care appointments, we will not provide you with any information.

After each intervention appointment, we will ask you to provide feedback to the research team. You may provide feedback in any format that is convenient for you (oral, written, email, Epic™ staff message, etc.). Additionally, at the end of the study, we may ask to interview you regarding topics such as: your overall perceptions, feasibility, shared decision-making with patients, thoughts about the written materials and future implementation. The interview is optional; you do not have to complete an interview. We anticipate that your time commitment will be as follows: about 10 minutes per patient during office visits, about 2 minutes per patient for after-visit feedback, and about 30 minutes for the end-of-study interview (if you choose to do so).

If you choose to provide oral feedback or participate in an end-of-study interview, we would like to audio record it, to ensure that we accurately capture your perspectives.

© 2021 Taksler GB et al. *JAMA Network Open.*

For both intervention and control appointments, your patients may be told that you are participating in a research study. We may also ask your patients (both intervention and usual care) to participate, in 2 ways. First, we may ask for feedback to help improve the study. We will only share aggregate feedback with you (no individual patient feedback). Second, we may review patient medical records, to see which health care services they receive within 1 year after their appointment. This will help us find out whether different ways to discuss health care eventually change patients' health.

What are the benefits to taking part in this study?
You may be able to help improve patient adherence to preventive care recommendations. Participating in this study may help researchers to gain further knowledge about what patients think about different things they can do to improve their health.

Are there any risks to you in participating in this study?
There are no physical risks associated with this study. If discussion of the written materials takes longer than anticipated, you may be late for other patient appointments. Some of the interview questions may make you feel uncomfortable or cause psychological stress or fatigue. You may refuse to answer any of the questions.

There is a potential risk of loss of confidentiality of your data. Every effort will be made to keep your information confidential through the following safeguards: All data will be stored in password-protected and encrypted storage facilities accessible only by the research team. No one with direct supervisory responsibilities for your work will have access to your individual responses. Participant names will be replaced by a study number so that individuals who do not need to know your name will not be able to connect your name with the research data. Only unique study identification numbers will identify participants in research databases. All data will be reported in aggregate and will not be linked to specific providers. Identifying information will be removed during the analysis and your responses will only be identified using an ID code.

Do I have to participate in the research?
Your participation is strictly voluntary. Your decision to participate will not impact your current or future employment a Cleveland Clinic or your performance reviews. The decision to participate or not to participate in our study will not affect your employment status in any way.

You may choose not to take part or may leave the study at any time. Leaving the study will not result in any penalty.

What other options are there?
Your participation is strictly voluntary. The alternative is to not participate.

What are the costs?
There is no cost for participation. You will not be compensated for participation in the study. Please be aware that, if your patients choose to provide feedback, they will be compensated $25.

Who do I contact if I have questions about the research?

© 2021 Taksler GB et al. *JAMA Network Open.*

If you have questions about the study after you have agreed, you may ask the study coordinator Jackie Fox, RN for assistance at any time (216-444-4590) or you can contact Glen Taksler, PhD (principal investigator) at 216-445-7499. If you have any questions about your rights as a research subject, you can contact the Institutional Review Board at (216) 444-2924.

Providing the study's written materials to a patient, discussing the written materials with a patient, providing after-visit feedback to the research team, and/or completion of the study interview will indicate your agreement to participate in the research.

Version 1.29.18

© 2021 Taksler GB et al. *JAMA Network Open.*

Final Patient Survey

#	Variable / Field Name	Field Label<br>*Field Note*	Field Attributes (Field Type, Validation, Choices, Calculations, etc.)
Instrument: **Patient Survey** (patient_survey) Enabled as survey Collapse			
1	record_id	Record ID	text
2	consent	Section Header: *You are being invited to take part in a research study by Cleveland Clinic. By participating in this study, you can help Cleveland Clinic researchers understand different ways to discuss health with patients. What are the requirements and time commitment to participate? You will be asked to complete a survey. The survey will take about 10-15 minutes. Also, we will review your medical record to see what health care services you receive within 1 year after your recent visit. This will help us find out whether different ways to discuss health care eventually change patients' health. What are the benefits of taking part in this study? There is no direct benefit to you from taking the survey. Participating in this study may help researchers gain further knowledge about the best ways to discuss health care services with patients. Are there any risks to you participating in this study? This is minimal risk*	radio<br>1 YES, I AGREE TO PARTICIPATE BY ANSWERING THIS SURVEY<br>2 NO, I DO NOT AGREE TO PARTICIPATE<br><br>Custom alignment: LV<br>Stop actions on 2

© 2021 Taksler GB et al. *JAMA Network Open.*

		research. You may experience anxiety or discomfort from answering survey questions. There is minimal risk to the confidentiality of your information. Do I have to participate in the research? Your participation is strictly voluntary. If you choose to participate, you can withdraw at any time. Will my privacy and confidentiality be protected? All of your data will be confidential. Your answers will NOT be shared with your doctor. What are the costs? There is no cost for you to participate. Will I be paid for my participation? Yes, you will receive a $25 gift card in appreciation of your time. How do I indicate that I want to participate in today's survey? To answer the survey, click "Yes, I agree to participate by answering this survey" at the bottom of this screen. Who do I contact if I have questions about the research? If you have any questions about this survey, you may contact the investigative team at (216) 444-4590. Or, if you have questions about your rights as a research participant, call the Cleveland Clinic Institutional Review Board at (216) 444-2924. Your help means a lot to us. We thank	

		you again for taking the time to complete this survey.<br>Do you consent to completing this survey?	
3	time_start	Section Header:<br>Start time	text (datetime_seconds_ymd)<br>Custom alignment: LV<br>Field Annotation: @NOW @HIDDEN
4	sex	What is your sex?	radio, Required<br>1 Male<br>2 Female
5	age	How old are you?	text, Required
6	ageminus10	Age minus 10	calc<br>Calculation: [age]-10<br>Custom alignment: LV<br>Field Annotation: @HIDDEN
7	ageminus1	Age minus 1	calc<br>Calculation: [age]-1<br>Custom alignment: LV<br>Field Annotation: @HIDDEN
8	ageplus1	Age plus 1	calc<br>Calculation: [age]+1<br>Custom alignment: LV<br>Field Annotation: @HIDDEN
9	ageplus2	Age plus 2	calc<br>Calculation: [age]+2<br>Custom alignment: LV<br>Field Annotation: @HIDDEN
10	ageplus3	Age plus 3	calc<br>Calculation: [age]+3<br>Custom alignment: LV<br>Field Annotation: @HIDDEN
11	ageplus5	Age plus 5	calc<br>Calculation: [age]+5<br>Custom alignment: LV<br>Field Annotation: @HIDDEN
12	ageplus6	Age plus 6	calc<br>Calculation: [age]+6<br>Custom alignment: LV<br>Field Annotation: @HIDDEN
13	ageplus9	Age plus 9	calc<br>Calculation: [age]+9<br>Custom alignment: LV<br>Field Annotation: @HIDDEN

© 2021 Taksler GB et al. *JAMA Network Open.*

14	ageplus10	Age plus 10	calc<br>Calculation: [age]+10<br>Custom alignment: LV<br>Field Annotation: @HIDDEN
15	discuss_alc	Section Header: *Please think back to today's visit with your doctor (or other health care professional). When answering these questions, please only think back to today's visit. Which of the following things did your doctor (or other health care professional) talk about today?*<br>Drink less alcohol	radio (Matrix), Required<br>1 Yes<br>2 No<br>3 Don't Know
16	discuss_dietex	Eat a healthy diet and exercise	radio (Matrix), Required<br>1 Yes<br>2 No<br>3 Don't Know
17	dsicuss_cholesterol_test	Get a cholesterol test	radio (Matrix), Required<br>1 Yes<br>2 No<br>3 Don't Know
18	discuss_diabetes	Get a diabetes test	radio (Matrix), Required<br>1 Yes<br>2 No<br>3 Don't Know
19	discuss_vaccine	Get a vaccine(for flu, pneumonia, etc)	radio (Matrix), Required<br>1 Yes<br>2 No<br>3 Don't Know
20	discuss_weight	Lose weight	radio (Matrix), Required<br>1 Yes<br>2 No

			3 Don't Know
21	discuss_bloodpressure	Lower my blood pressure	radio (Matrix), Required<br>1 Yes<br>2 No<br>3 Don't Know
22	discuss_bloodsugar	Lower my blood sugar	radio (Matrix), Required<br>1 Yes<br>2 No<br>3 Don't Know
23	discuss_cholesterol	Lower my cholesterol	radio (Matrix), Required<br>1 Yes<br>2 No<br>3 Don't Know
24	discuss_aneurysm	Check for an aneurysm	radio (Matrix), Required<br>1 Yes<br>2 No<br>3 Don't Know
25	discuss_breastcancer<br>Show the field ONLY if:<br>[sex] = '2'	Check for breast cancer(Get a mammogram)	radio (Matrix), Required<br>1 Yes<br>2 No<br>3 Don't Know
26	discuss_cervicalcancer<br>Show the field ONLY if:<br>[sex] = '2'	Check for cervical cancer(Get a PAP test)	radio (Matrix), Required<br>1 Yes<br>2 No<br>3 Don't Know
27	discuss_colorectal	Check for colon cancer(Get a colonoscopy or atest for blood in your stool)	radio (Matrix), Required<br>1 Yes<br>2 No<br>3 Don't Know
28	discuss_lung	Check for lung cancer	radio (Matrix), Required<br>1 Yes

© 2021 Taksler GB et al. *JAMA Network Open.*

			2 No<br>3 Don't Know
29	discuss_aspirin	Take aspirin	radio (Matrix), Required<br>1 Yes<br>2 No<br>3 Don't Know
30	discuss_std	Test for a sexually transmitted disease	radio (Matrix), Required<br>1 Yes<br>2 No<br>3 Don't Know
31	discuss_smoking	Quit smoking	radio (Matrix), Required<br>1 Yes<br>2 No<br>3 Don't Know
32	rec_alc<br>Show the field ONLY if:<br>[discuss_alc] = '1'	Section Header: *We have been asking you about preventive care services. Many of these things will not improve your health immediately, but can help you to be healthier as you get older, and to live longer. When doctors (or other health care professionals) discuss preventive care services, they can recommend that you do something, or that you NOT do something. For example, if you recently had a cholesterol test, then your doctor may not recommend another test right now. At today's visit, what did your doctor (or other health care*	radio (Matrix), Required<br>1 My doctor recommended this<br>2 My doctor did NOT recommend this<br>3 Don't Know

© 2021 Taksler GB et al. *JAMA Network Open.*

		professional) recommend for each of the following preventive services?<br>Drink less alcohol	
33	rec_dietex<br>Show the field ONLY if:<br>[discuss_dietex] = '1'	Eat a healthy diet and exercise	radio (Matrix), Required<br>1 My doctor recommended this<br>2 My doctor did NOT recommend this<br>3 Don't Know
34	rec_cholesterol_test<br>Show the field ONLY if:<br>[dsicuss_cholesterol_test] = '1'	Get a cholesterol test	radio (Matrix), Required<br>1 My doctor recommended this<br>2 My doctor did NOT recommend this<br>3 Don't Know
35	rec_diabetes<br>Show the field ONLY if:<br>[discuss_diabetes] = '1'	Get a diabetes test	radio (Matrix), Required<br>1 My doctor recommended this<br>2 My doctor did NOT recommend this<br>3 Don't Know
36	rec_vaccine<br>Show the field ONLY if:<br>[discuss_vaccine] = '1'	Get a vaccine (for flu, pneumonia, etc)	radio (Matrix), Required<br>1 My doctor recommended this<br>2 My doctor did NOT recommend this<br>3 Don't Know
37	rec_weight<br>Show the field ONLY if:<br>[discuss_weight] = '1'	Lose weight	radio (Matrix), Required<br>1 My doctor recommended this<br>2 My doctor did NOT recommend this<br>3 Don't Know
38	rec_bloodpressure<br>Show the field ONLY if:<br>[discuss_bloodpressure] = '1'	Lower my blood pressure	radio (Matrix), Required<br>1 My doctor recommended this<br>2 My doctor did NOT recommend this<br>3 Don't Know
39	rec_bloodsugar<br>Show the field ONLY if:<br>[discuss_bloodsugar] = '1'	Lower my blood sugar	radio (Matrix), Required<br>1 My doctor recommended this<br>2 My doctor did NOT recommend this

© 2021 Taksler GB et al. *JAMA Network Open.*

			3 Don't Know
40	rec_cholesterol<br>Show the field ONLY if:<br>[discuss_cholesterol] = '1'	Lower my cholesterol	radio (Matrix), Required<br>1 My doctor recommended this<br>2 My doctor did NOT recommend this<br>3 Don't Know
41	rec_aneurysm<br>Show the field ONLY if:<br>[discuss_aneurysm]='1'	Check for an aneurysm	radio (Matrix), Required<br>1 My doctor recommended this<br>2 My doctor did NOT recommend this<br>3 Don't Know
42	rec_breastcancer<br>Show the field ONLY if:<br>[discuss_breastcancer]='1' and [sex]='2'	Check for breast cancer(Get a mammogram)	radio (Matrix), Required<br>1 My doctor recommended this<br>2 My doctor did NOT recommend this<br>3 Don't Know
43	rec_cervicalcancer<br>Show the field ONLY if:<br>[discuss_cervicalcancer]='1' a nd [sex]='2'	Check for cervical cancer(Get a PAP test)	radio (Matrix), Required<br>1 My doctor recommended this<br>2 My doctor did NOT recommend this<br>3 Don't Know
44	rec_colorectal<br>Show the field ONLY if:<br>[discuss_colorectal]='1'	Check for colon cancer(Get a colonoscopy or atest for blood in your stool)	radio (Matrix), Required<br>1 My doctor recommended this<br>2 My doctor did NOT recommend this<br>3 Don't Know
45	rec_lung<br>Show the field ONLY if:<br>[discuss_lung]='1'	Check for lung cancer	radio (Matrix), Required<br>1 My doctor recommended this<br>2 My doctor did NOT recommend this<br>3 Don't Know
46	rec_aspirin<br>Show the field ONLY if:<br>[discuss_aspirin]='1'	Take aspirin	radio (Matrix), Required<br>1 My doctor recommended this<br>2 My doctor did NOT recommend this<br>3 Don't Know
47	rec_std<br>Show the field ONLY if:<br>[discuss_std]='1'	Test for a sexually transmitted disease	radio (Matrix), Required<br>1 My doctor recommended this

<table>
<tr><td></td><td></td><td></td><td><table><tr><td>2</td><td>My doctor did NOT recommend this</td></tr><tr><td>3</td><td>Don't Know</td></tr></table></td></tr>
<tr><td>48</td><td>rec_smoking
Show the field ONLY if:
[discuss_smoking]='1'</td><td>Quit smoking</td><td>radio (Matrix), Required<table><tr><td>1</td><td>My doctor recommended this</td></tr><tr><td>2</td><td>My doctor did NOT recommend this</td></tr><tr><td>3</td><td>Don't Know</td></tr></table></td></tr>
<tr><td>49</td><td>graphsold</td><td>Section Header:
At today's visit, did your doctor (or other health care professional) show you a graph with the title "How Can You Improve Your Health?"

If you didn't see the graph, that's ok. About half of patients saw the graph, and the other half didn't see it.

For this question, we are only asking about a graph with the title "How Can You Improve Your Health?"</td><td>radio<table><tr><td>1</td><td>Yes</td></tr><tr><td>0</td><td>No</td></tr></table>
Custom alignment: LV
Field Annotation: @HIDDEN</td></tr>
<tr><td>50</td><td>hidgraph</td><td>At today's visit, did your doctor (or other health care professional) show you a graph with the title "How Can You Improve Your Health?"

It would have looked similar to this:</td><td>descriptive</td></tr>
</table>

© 2021 Taksler GB et al. *JAMA Network Open.*

<table>
<tr><td>51</td><td>graphs</td><td>If you didn't see the graph, that's ok. About half of patients saw the graph, and other other half didn't see it.

At today's visit, did your doctor (or other health care professional) show you a graph with the title "How Can You Improve Your Health?"</td><td>radio, Required
1 Yes
0 No

Custom alignment: LV</td></tr>
<tr><td>52</td><td>handout
Show the field ONLY if:
[graphs] = '1'</td><td>Section Header:
Was the graph with the title "How Can You Improve Your Health" written (a handout), on a computer or both?</td><td>radio, Required
1 Written (a handout)
2 On a computer
3 Both

Custom alignment: LV</td></tr>
<tr><td>53</td><td>helpful_information
Show the field ONLY if:
[graphs] = '1'</td><td>How helpful did you find the "How Can You Improve Your Health" information?</td><td>radio (Matrix), Required
1 Not at all helpful1
2 2
3 3
4 4
5 5
6 6
7 7
8 8
9 9
10 Extremely Helpful10</td></tr>
<tr><td>54</td><td>own_words_best
Show the field ONLY if:
[graphs] = '1'</td><td>In your own words, what did you like BEST about the "How Can You Improve Your</td><td>notes, Required</td></tr>
</table>

© 2021 Taksler GB et al. *JAMA Network Open.*

		Health" information?	
55	own_words_least<br>Show the field ONLY if:<br>[graphs] = '1'	In your own words, what did you like LEAST about the "How Can You Improve Your Health" information?	notes, Required
56	updated_information<br>Show the field ONLY if:<br>[graphs] = '1'	In the future, would you like to see updated "How Can You Improve Your Health" information?	radio (Matrix), Required<br>1 Not at all interested1<br>2 2<br>3 3<br>4 4<br>5 5<br>6 6<br>7 7<br>8 8<br>9 9<br>10 Extremely interested10
57	hidheader_i<br>Show the field ONLY if:<br>[graphs] = '1'	Section Header:<br>We would now like to ask you more about the graph with the title "How Can You Improve Your Health". Please take another look at the graph, and then use it to answer the following questions.<br><br>If you don't know, please just take your best guess.	descriptive
58	hidheader_c<br>Show the field ONLY if:<br>[graphs] = '0'	Please answer the following questions based on today's visit.	descriptive

© 2021 Taksler GB et al. *JAMA Network Open.*

		If you don't know, please just take your best guess.	
59	hidcurrage_intro_i<br>Show the field ONLY if:<br>[graphs]='1'	Section Header:<br>People age [age] years old have a wide range of health conditions.Some people are in very good health and have the health of a [ageminus10] year old.Other people are in worse health and have the health of a [ageplus10] year old.<br><br>Based on the "How Can You Improve Your Health" information, do you have the health of someone who is: (Choose one)	descriptive
60	hidcurrage_intro_c<br>Show the field ONLY if:<br>[graphs]='0'	People age [age] years old have a wide range of health conditions.<br>Some people are in very good health and have the health of a [ageminus10] year old.<br>Other people are in worse health and have the health of a [ageplus10] year old.<br><br>Based on today's visit, do you have the	descriptive

© 2021 Taksler GB et al. *JAMA Network Open.*

		health of someone who is: (Choose one)	
61	currage		radio, Required<br>1 [ageminus1] years old or younger<br>2 Similar to other [age] year olds<br>3 [ageplus1] to [ageplus2] years old<br>4 [ageplus3] to [ageplus5] years old<br>5 [ageplus6] to [ageplus9] years old<br>6 [ageplus10] years old or older<br><br>Custom alignment: LV
62	hidcurrage_guess	If you don't know, please just take your best guess.	descriptive
63	hidmost_i<br>Show the field ONLY if:<br>[graphs]='1'	Section Header:<br>People can improve their health in different ways. Based on the "How Can You Improve Your Health" information, which of the following preventive services is MOST likely to improve your health?<br><br>If you don't know, please just take your best guess. (Choose one)	descriptive
64	hidmost_c<br>Show the field ONLY if:<br>[graphs]='0'	People can improve their health in different ways. Based on today's visit, which of the following preventive	descriptive

© 2021 Taksler GB et al. *JAMA Network Open.*

		services is MOST likely to improve your health?<br><br>If you don't know, please just take your best guess. (Choose one)	
65	most_alcohol<br>Show the field ONLY if:<br>[rec_alc] = '1'	Drink less alcohol	radio (Matrix - ranking)<br>1 MOST likely to improve my health
66	most_dietex<br>Show the field ONLY if:<br>[rec_dietex]='1'	Eat a healthy diet and exercise	radio (Matrix - ranking)<br>1 MOST likely to improve my health
67	most_cholesterol<br>Show the field ONLY if:<br>[rec_cholesterol_test]='1'	Get a cholesterol test	radio (Matrix - ranking)<br>1 MOST likely to improve my health
68	most_diabetes<br>Show the field ONLY if:<br>[rec_diabetes]='1'	Get a diabetes test	radio (Matrix - ranking)<br>1 MOST likely to improve my health
69	most_vaccine<br>Show the field ONLY if:<br>[rec_vaccine]='1'	Get a vaccine (for flu, pneumonia, etc)	radio (Matrix - ranking)<br>1 MOST likely to improve my health
70	most_lose_weight<br>Show the field ONLY if:<br>[rec_weight]='1'	Lose weight	radio (Matrix - ranking)<br>1 MOST likely to improve my health
71	most_blood_pressure<br>Show the field ONLY if:<br>[rec_bloodpressure]='1'	Lower my blood pressure	radio (Matrix - ranking)<br>1 MOST likely to improve my health
72	most_blood_sugar<br>Show the field ONLY if:<br>[rec_bloodsugar]='1'	Lower my blood sugar	radio (Matrix - ranking)<br>1 MOST likely to improve my health
73	most_lower_cholesterol<br>Show the field ONLY if:<br>[rec_cholesterol]='1'	Lower my cholesterol	radio (Matrix - ranking)<br>1 MOST likely to improve my health
74	most_aneurysm<br>Show the field ONLY if:<br>[rec_aneurysm]='1'	Check for an aneurysm	radio (Matrix - ranking)<br>1 MOST likely to improve my health
75	most_breast<br>Show the field ONLY if:<br>[rec_breastcancer]='1' and [sex]='2'	Check for breast cancer(Get a mammogram)	radio (Matrix - ranking)<br>1 MOST likely to improve my health
76	most_cervical<br>Show the field ONLY if:<br>[rec_cervicalcancer]='1' and [sex]='2'	Check for cervical cancer(Get a PAP test)	radio (Matrix - ranking)<br>1 MOST likely to improve my health

© 2021 Taksler GB et al. *JAMA Network Open.*

77	most_colon<br>Show the field ONLY if:<br>[rec_colorectal]='1'	Check for colon cancer(Get a colonoscopy or atest for blood in your stool)	radio (Matrix - ranking)<br>1 MOST likely to improve my health
78	most_lung<br>Show the field ONLY if:<br>[rec_lung]='1'	Check for lung cancer	radio (Matrix - ranking)<br>1 MOST likely to improve my health
79	most_aspirin<br>Show the field ONLY if:<br>[rec_aspirin]='1'	Take aspirin	radio (Matrix - ranking)<br>1 MOST likely to improve my health
80	most_std<br>Show the field ONLY if:<br>[rec_std]='1'	Test for a sexually transmitted disease	radio (Matrix - ranking)<br>1 MOST likely to improve my health
81	most_smoking<br>Show the field ONLY if:<br>[rec_smoking]='1'	Quit smoking	radio (Matrix - ranking)<br>1 MOST likely to improve my health
82	most_younger<br>Show the field ONLY if:<br>[graphs]='1'	Section Header:<br>If you do that, will you have the health of someone who is:	radio, Required<br>1 1 month to 6 months younger<br>2 7 months to 11 months younger<br>3 1 year to 2 years younger<br>4 3 years to 5 years younger<br>5 6 years to 9 years younger<br>6 10 years or more younger<br><br>Custom alignment: LV
83	hidmost_guess<br>Show the field ONLY if:<br>[graphs]='1'	If you don't know, please just take your best guess.	descriptive
84	hidleast_i<br>Show the field ONLY if:<br>[graphs]='1'	Section Header:<br>All preventive services are important. But, some are more likely to improve your health than others. Based on the "How Can You Improve Your Health" information, which of the following	descriptive

© 2021 Taksler GB et al. *JAMA Network Open.*

		preventive services is LEAST likely to improve your health?<br><br>If you don't know, please just take your best guess. (Choose one)	
85	hidleast_c<br>Show the field ONLY if:<br>[graphs]='0'	All preventive services are important. But, some are more likely to improve your health than others. Based on today's visit, which of the following preventive services is LEAST likely to improve your health?<br><br>If you don't know, please just take your best guess. (Choose one)	descriptive
86	least_alcohol<br>Show the field ONLY if:<br>[rec_alc]='1' and [most_alcohol]!='1'	Drink less alcohol	radio (Matrix - ranking)<br>1 LEAST likely to improve my health
87	least_dietex<br>Show the field ONLY if:<br>[rec_dietex]='1' and [most_dietex]!='1'	Eat a healthy diet and exercise	radio (Matrix - ranking)<br>1 LEAST likely to improve my health
88	least_cholesterol<br>Show the field ONLY if:<br>[rec_cholesterol_test]='1' and [most_cholesterol]!='1'	Get a cholesterol test	radio (Matrix - ranking)<br>1 LEAST likely to improve my health
89	least_diabetes<br>Show the field ONLY if:<br>[rec_diabetes]='1' and [most_diabetes]!='1'	Get a diabetes test	radio (Matrix - ranking)<br>1 LEAST likely to improve my health
90	least_vaccine<br>Show the field ONLY if:	Get a vaccine(for flu, pneumonia, etc.)	radio (Matrix - ranking)<br>1 LEAST likely to improve my health

© 2021 Taksler GB et al. *JAMA Network Open.*

	[rec_vaccine]='1' and [most_vaccine]!='1'		
91	least_lose_weight<br>Show the field ONLY if:<br>[rec_weight]='1' and [most_lose_weight]!='1'	Lose weight	radio (Matrix - ranking)<br>1 LEAST likely to improve my health
92	least_blood_pressure<br>Show the field ONLY if:<br>[rec_bloodpressure]='1' and [most_blood_pressure]!='1'	Lower my blood pressure	radio (Matrix - ranking)<br>1 LEAST likely to improve my health
93	least_blood_sugar<br>Show the field ONLY if:<br>[rec_bloodsugar]='1' and [most_blood_sugar]!='1'	Lower my blood sugar	radio (Matrix - ranking)<br>1 LEAST likely to improve my health
94	least_lower_cholesterol<br>Show the field ONLY if:<br>[rec_cholesterol]='1' and [most_lower_cholesterol]!='1'	Lower my cholesterol	radio (Matrix - ranking)<br>1 LEAST likely to improve my health
95	least_aneurysm<br>Show the field ONLY if:<br>[rec_aneurysm]='1' and [most_aneurysm]!='1'	Check for an aneurysm	radio (Matrix - ranking)<br>1 LEAST likely to improve my health
96	least_breast<br>Show the field ONLY if:<br>[rec_breastcancer]='1' and [sex]='2' and [most_breast]!='1'	Check for breast cancer(Get a mammogram)	radio (Matrix - ranking)<br>1 LEAST likely to improve my health
97	least_cervical<br>Show the field ONLY if:<br>[rec_cervicalcancer]='1' and [sex]='2' and [most_cervical]!='1'	Check for cervical cancer(Get a PAP test)	radio (Matrix - ranking)<br>1 LEAST likely to improve my health
98	least_colon<br>Show the field ONLY if:<br>[rec_colorectal]='1' and [most_colon]!='1'	Check for colon cancer(Get a colonoscopy or test for blood in your stool)	radio (Matrix - ranking)<br>1 LEAST likely to improve my health
99	least_lung<br>Show the field ONLY if:<br>[rec_lung]='1' and [most_lung]!='1'	Check for lung cancer	radio (Matrix - ranking)<br>1 LEAST likely to improve my health
100	least_aspirin<br>Show the field ONLY if:<br>[rec_aspirin]='1' and [most_aspirin]!='1'	Take aspirin	radio (Matrix - ranking)<br>1 LEAST likely to improve my health
101	least_std<br>Show the field ONLY if:<br>[rec_std]='1' and [most_std]!='1'	Test for a sexually transmitted disease	radio (Matrix - ranking)<br>1 LEAST likely to improve my health

© 2021 Taksler GB et al. *JAMA Network Open.*

102	least_smoking<br>Show the field ONLY if:<br>[rec_smoking]='1' and [most_smoking]!='1'	Quit smoking	radio (Matrix - ranking)<br>1 LEAST likely to improve my health
103	least_younger<br>Show the field ONLY if:<br>[graphs]='1'	Section Header:<br>If you do that, will you have the health of someone who is:	radio, Required<br>1 1 month to 6 months younger<br>2 7 months to 11 months younger<br>3 1 year to 2 years younger<br>4 3 years to 5 years younger<br>5 6 years to 9 years younger<br>6 10 years or more younger<br><br>Custom alignment: LV
104	hidleast_guess<br>Show the field ONLY if:<br>[graphs]='1'	If you don't know, please just take your best guess.	descriptive
105	hidleast_thank	Thank you!	descriptive
106	hidimpt_i<br>Show the field ONLY if:<br>[graphs]='1'	Section Header:<br>Based on the "How Can You Improve Your Health" information, are the following preventive services likely to improve your health?<br><br>You can use a scale from "Not at all likely" to "Very likely."<br><br>If you don't know, please just take your best guess.	descriptive
107	hidimpt_c<br>Show the field ONLY if:<br>[graphs]='0'	Based on today's visit, are the following preventive services likely to improve your health?	descriptive

		You can use a scale from "Not at all likely" to "Very likely."<br><br>If you don't know, please just take your best guess.	
108	impt_alcohol<br>Show the field ONLY if:<br>[rec_alc] = '1'	Drink less alcohol	radio (Matrix), Required<br>1 Not at all likely1<br>2 2<br>3 3<br>4 4<br>5 5<br>6 6<br>7 Very likely7
109	impt_dietex<br>Show the field ONLY if:<br>[rec_dietex] = '1'	Eat a healthy diet and exercise	radio (Matrix), Required<br>1 Not at all likely1<br>2 2<br>3 3<br>4 4<br>5 5<br>6 6<br>7 Very likely7
110	impt_cholesterol<br>Show the field ONLY if:<br>[rec_cholesterol_test] = '1'	Get a cholesterol test	radio (Matrix), Required<br>1 Not at all likely1<br>2 2<br>3 3<br>4 4<br>5 5<br>6 6<br>7 Very likely7
111	impt_diabetes<br>Show the field ONLY if:<br>[rec_diabetes] = '1'	Get a diabetes test	radio (Matrix), Required<br>1 Not at all likely1

© 2021 Taksler GB et al. *JAMA Network Open.*

			2 2<br>3 3<br>4 4<br>5 5<br>6 6<br>7 Very likely7
112	impt_vaccine<br>Show the field ONLY if:<br>[rec_vaccine] = '1'	Get a vaccine (for flu, pneumonia, etc)	radio (Matrix), Required<br>1 Not at all likely1<br>2 2<br>3 3<br>4 4<br>5 5<br>6 6<br>7 Very likely7
113	impt_lose_weight<br>Show the field ONLY if:<br>[rec_weight] = '1'	Lose weight	radio (Matrix), Required<br>1 Not at all likely1<br>2 2<br>3 3<br>4 4<br>5 5<br>6 6<br>7 Very likely7
114	impt_blood_pressure<br>Show the field ONLY if:<br>[rec_bloodpressure] = '1'	Lower my blood pressure	radio (Matrix), Required<br>1 Not at all likely1<br>2 2<br>3 3<br>4 4<br>5 5<br>6 6<br>7 Very likely7

© 2021 Taksler GB et al. *JAMA Network Open.*

115	impt_blood_sugar<br>Show the field ONLY if:<br>[rec_bloodsugar] = '1'	Lower my blood sugar	radio (Matrix), Required<br>1 Not at all likely1<br>2 2<br>3 3<br>4 4<br>5 5<br>6 6<br>7 Very likely7
116	impt_lower_cholesterol<br>Show the field ONLY if:<br>[rec_cholesterol] = '1'	Lower my cholesterol	radio (Matrix), Required<br>1 Not at all likely1<br>2 2<br>3 3<br>4 4<br>5 5<br>6 6<br>7 Very likely7
117	impt_aneurysm<br>Show the field ONLY if:<br>[rec_aneurysm] = '1'	Check for an aneurysm	radio (Matrix), Required<br>1 Not at all likely1<br>2 2<br>3 3<br>4 4<br>5 5<br>6 6<br>7 Very likely7
118	impt_breast<br>Show the field ONLY if:<br>[sex] = '2' and [rec_breastcancer] = '1'	Check for breast cancer(Get a mammogram)	radio (Matrix), Required<br>1 Not at all likely1<br>2 2<br>3 3<br>4 4<br>5 5<br>6 6

			7 Very likely7
119	impt_cervical<br>Show the field ONLY if:<br>[sex] = '2' and [rec_cervicalcancer] = '1'	Check for cervical cancer(Get a PAP test)	radio (Matrix), Required<br>1 Not at all likely1<br>2 2<br>3 3<br>4 4<br>5 5<br>6 6<br>7 Very likely7
120	impt_colon<br>Show the field ONLY if:<br>[rec_colorectal] = '1'	Check for colon cancer(Get a colonoscopy or atest for blood in your stool)	radio (Matrix), Required<br>1 Not at all likely1<br>2 2<br>3 3<br>4 4<br>5 5<br>6 6<br>7 Very likely7
121	impt_lung<br>Show the field ONLY if:<br>[rec_lung] = '1'	Check for lung cancer	radio (Matrix), Required<br>1 Not at all likely1<br>2 2<br>3 3<br>4 4<br>5 5<br>6 6<br>7 Very likely7
122	impt_aspirin<br>Show the field ONLY if:<br>[rec_aspirin] = '1'	Take aspirin	radio (Matrix), Required<br>1 Not at all likely1<br>2 2<br>3 3<br>4 4<br>5 5

© 2021 Taksler GB et al. *JAMA Network Open.*

			6 6<br>7 Very likely7
123	impt_std<br>Show the field ONLY if:<br>[rec_std] = '1'	Test for a sexually transmitted disease	radio (Matrix), Required<br>1 Not at all likely1<br>2 2<br>3 3<br>4 4<br>5 5<br>6 6<br>7 Very likely7
124	impt_smoking<br>Show the field ONLY if:<br>[rec_smoking] = '1'	Quit smoking	radio (Matrix), Required<br>1 Not at all likely1<br>2 2<br>3 3<br>4 4<br>5 5<br>6 6<br>7 Very likely7
125	hidintro_decision	Section Header:<br>The next set of questions asks about your conversation with your doctor (or other health care professional) today.<br><br>As a reminder, all of your data will be confidential. You answers will NOT be shared with your doctor (or other health care professional).	descriptive

© 2021 Taksler GB et al. *JAMA Network Open.*

126	decision_made	Section Header: *Decisions about preventive care services can be made in many different ways. We would like to learn more about how you and your doctor (or other health care professional) talked about these decisions, during your appointment today. You can use a scale from "Completely Disagree" to "Completely Agree."*<br><br>My doctor made clear that a decision needs to be made about my preventive care.	radio (Matrix), Required<br>1 Completely Disagree1<br>2 Strongly Disagree2<br>3 Somewhat Disagree3<br>4 Somewhat Agree4<br>5 Strongly Agree5<br>6 Completely Agree6
127	decision_involved	My doctor wanted to know exactly how I want to be involved in making a decision about my preventive care.	radio (Matrix), Required<br>1 Completely Disagree1<br>2 Strongly Disagree2<br>3 Somewhat Disagree3<br>4 Somewhat Agree4<br>5 Strongly Agree5<br>6 Completely Agree6
128	decision_options	My doctor told me there are different options for preventive care.	radio (Matrix), Required<br>1 Completely Disagree1<br>2 Strongly Disagree2<br>3 Somewhat Disagree3<br>4 Somewhat Agree4<br>5 Strongly Agree5<br>6 Completely Agree6
129	decision_ad_dis	My doctor precisely explained the advantages and disadvantages of	radio (Matrix), Required<br>1 Completely Disagree1<br>2 Strongly Disagree2

© 2021 Taksler GB et al. *JAMA Network Open.*

		the preventive care options.	3 Somewhat Disagree3<br>4 Somewhat Agree4<br>5 Strongly Agree5<br>6 Completely Agree6
130	decision_understand	My doctor helped me understand all the information.	radio (Matrix), Required<br>1 Completely Disagree1<br>2 Strongly Disagree2<br>3 Somewhat Disagree3<br>4 Somewhat Agree4<br>5 Strongly Agree5<br>6 Completely Agree6
131	decision_preference	My doctor asked me which preventive care options I prefer.	radio (Matrix), Required<br>1 Completely Disagree1<br>2 Strongly Disagree2<br>3 Somewhat Disagree3<br>4 Somewhat Agree4<br>5 Strongly Agree5<br>6 Completely Agree6
132	decision_weighted	My doctor and I thoroughly weighed the different preventive care options.	radio (Matrix), Required<br>1 Completely Disagree1<br>2 Strongly Disagree2<br>3 Somewhat Disagree3<br>4 Somewhat Agree4<br>5 Strongly Agree5<br>6 Completely Agree6
133	decisions_selected	My doctor and I selected preventive care options together.	radio (Matrix), Required<br>1 Completely Disagree1<br>2 Strongly Disagree2<br>3 Somewhat Disagree3<br>4 Somewhat Agree4<br>5 Strongly Agree5

© 2021 Taksler GB et al. *JAMA Network Open.*

			6 Completely Agree6
134	decision_agreement	My doctor and I reached an agreement on how to proceed.	radio (Matrix), Required<br>1 Completely Disagree1<br>2 Strongly Disagree2<br>3 Somewhat Disagree3<br>4 Somewhat Agree4<br>5 Strongly Agree5<br>6 Completely Agree6
135	how_decide	Section Header:<br>Please choose the ONE statement that best describes how you would like to make a decision about which preventive care services are best for you.	radio, Required<br>1 I would like my doctor(s) to make the decisions with little input from me.<br>2 I would like my doctor(s) to make the decisions but to seriously consider my opinion.<br>3 I would like my doctors(s) and me to make the decisions together.<br>4 I would like to make the decisions after seriously considering my doctor(s) opinion.<br>5 I would like to make the decision with little input from my doctor(s).
136	thankyou	Thank you! We have about 5 minutes left.	descriptive
137	month_drinkless<br>Show the field ONLY if:<br>[rec_alc] = '1'	Section Header: *It can be hard when your doctor (or other health care professional) asks you to make a lot of changes to improve your health. When answering the following questions. please think about how many changes you could make in your life at once, while also maintaining your relationships with your family and friends, your work, and your*	radio (Matrix), Required<br>1 Not at all likely1<br>2 2<br>3 3<br>4 4<br>5 5<br>6 6<br>7 Very likely7

© 2021 Taksler GB et al. *JAMA Network Open.*

		hobbies. There are no "right" answers. In your opinion, how likely are you to do the following things in the next 1 month?<br>Drink less alcohol	
138	month_dietex<br>Show the field ONLY if:<br>[rec_dietex] = '1'	Eat a healthier diet and exercise	radio (Matrix), Required<br>1 Not at all likely1<br>2 2<br>3 3<br>4 4<br>5 5<br>6 6<br>7 Very likely7
139	month_cholesterol<br>Show the field ONLY if:<br>[rec_cholesterol_test] = '1'	Get a cholesterol test	radio (Matrix), Required<br>1 Not at all likely1<br>2 2<br>3 3<br>4 4<br>5 5<br>6 6<br>7 Very likely7
140	month_diabetes_test<br>Show the field ONLY if:<br>[rec_diabetes] = '1'	Get a diabetes test	radio (Matrix), Required<br>1 Not at all likely1<br>2 2<br>3 3<br>4 4<br>5 5<br>6 6<br>7 Very likely7
141	month_vaccine<br>Show the field ONLY if:<br>[rec_vaccine] = '1'	Get a vaccine (for flu, pneumonia, etc)	radio (Matrix), Required<br>1 Not at all likely1

© 2021 Taksler GB et al. *JAMA Network Open.*

			<table><tr><td>2</td><td>2</td></tr><tr><td>3</td><td>3</td></tr><tr><td>4</td><td>4</td></tr><tr><td>5</td><td>5</td></tr><tr><td>6</td><td>6</td></tr><tr><td>7</td><td>Very likely7</td></tr></table>
142	month_loss_weight<br>Show the field ONLY if:<br>[rec_weight] = '1'	Lose weight	radio (Matrix), Required<table><tr><td>1</td><td>Not at all likely1</td></tr><tr><td>2</td><td>2</td></tr><tr><td>3</td><td>3</td></tr><tr><td>4</td><td>4</td></tr><tr><td>5</td><td>5</td></tr><tr><td>6</td><td>6</td></tr><tr><td>7</td><td>Very likely7</td></tr></table>
143	month_lower_blood_pressure<br>Show the field ONLY if:<br>[rec_bloodpressure] = '1'	Lower my blood pressure	radio (Matrix), Required<table><tr><td>1</td><td>Not at all likely1</td></tr><tr><td>2</td><td>2</td></tr><tr><td>3</td><td>3</td></tr><tr><td>4</td><td>4</td></tr><tr><td>5</td><td>5</td></tr><tr><td>6</td><td>6</td></tr><tr><td>7</td><td>Very likely7</td></tr></table>
144	month_lower_blood_sugar<br>Show the field ONLY if:<br>[rec_bloodsugar] = '1'	Lower my blood sugar	radio (Matrix), Required<table><tr><td>1</td><td>Not at all likely1</td></tr><tr><td>2</td><td>2</td></tr><tr><td>3</td><td>3</td></tr><tr><td>4</td><td>4</td></tr><tr><td>5</td><td>5</td></tr><tr><td>6</td><td>6</td></tr><tr><td>7</td><td>Very likely7</td></tr></table>

145	month_lower_cholesterol<br>Show the field ONLY if:<br>[rec_cholesterol] = '1'	Lower my cholesterol	radio (Matrix), Required<br>1 Not at all likely1<br>2 2<br>3 3<br>4 4<br>5 5<br>6 6<br>7 Very likely7
146	month_aneurysm<br>Show the field ONLY if:<br>[rec_aneurysm] = '1'	Check for an aneurysm	radio (Matrix), Required<br>1 Not at all likely1<br>2 2<br>3 3<br>4 4<br>5 5<br>6 6<br>7 Very likely7
147	month_screen_breast<br>Show the field ONLY if:<br>[sex] = '2' and [rec_breastcancer] = '1'	Check for breast cancer(Get a mammogram)	radio (Matrix), Required<br>1 Not at all likely1<br>2 2<br>3 3<br>4 4<br>5 5<br>6 6<br>7 Very likely7
148	month_screen_cervical<br>Show the field ONLY if:<br>[sex] = '2' and [rec_cervicalcancer] = '1'	Check for cervical cancer(Get a PAP test)	radio (Matrix), Required<br>1 Not at all likely1<br>2 2<br>3 3<br>4 4<br>5 5<br>6 6

© 2021 Taksler GB et al. *JAMA Network Open.*

			7 Very likely7
149	month_screen_colorectal<br>Show the field ONLY if:<br>[rec_colorectal] = '1'	Check for colon cancer(Get a colonoscopy or atest for blood in your stool)	radio (Matrix), Required<br>1 Not at all likely1<br>2 2<br>3 3<br>4 4<br>5 5<br>6 6<br>7 Very likely7
150	month_screen_lung<br>Show the field ONLY if:<br>[rec_lung] = '1'	Check for lung cancer	radio (Matrix), Required<br>1 Not at all likely1<br>2 2<br>3 3<br>4 4<br>5 5<br>6 6<br>7 Very likely7
151	month_aspirin<br>Show the field ONLY if:<br>[rec_aspirin] = '1'	Take aspirin	radio (Matrix), Required<br>1 Not at all likely1<br>2 2<br>3 3<br>4 4<br>5 5<br>6 6<br>7 Very likely7
152	month_std<br>Show the field ONLY if:<br>[rec_std] = '1'	Test for a sexually transmitted disease	radio (Matrix), Required<br>1 Not at all likely1<br>2 2<br>3 3<br>4 4<br>5 5

© 2021 Taksler GB et al. *JAMA Network Open.*

			6 6<br>7 Very likely7
153	month_smoking<br>Show the field ONLY if:<br>[rec_smoking] = '1'	Quit smoking	radio (Matrix), Required<br>1 Not at all likely1<br>2 2<br>3 3<br>4 4<br>5 5<br>6 6<br>7 Very likely7
154	six_mon_alcohol<br>Show the field ONLY if:<br>[rec_alc] = '1'	Section Header: *In your opinion, how likely are you to do the following things in the next 6 months?*<br>Drink less alcohol	radio (Matrix), Required<br>1 Not at all likely1<br>2 2<br>3 3<br>4 4<br>5 5<br>6 6<br>7 Very likely7
155	six_mon_dietex<br>Show the field ONLY if:<br>[rec_dietex] = '1'	Eat a healthier diet and exercise	radio (Matrix), Required<br>1 Not at all likely1<br>2 2<br>3 3<br>4 4<br>5 5<br>6 6<br>7 Very likely7
156	six_mon_cholesterol<br>Show the field ONLY if:<br>[rec_cholesterol_test] = '1'	Get a cholesterol test	radio (Matrix), Required<br>1 Not at all likely1<br>2 2<br>3 3<br>4 4

© 2021 Taksler GB et al. *JAMA Network Open.*

			5 5<br>6 6<br>7 Very likely7
157	six_mon_test_diabetes<br>Show the field ONLY if:<br>[rec_diabetes] = '1'	Get a diabetes test	radio (Matrix), Required<br>1 Not at all likely1<br>2 2<br>3 3<br>4 4<br>5 5<br>6 6<br>7 Very likely7
158	six_mon_vaccine<br>Show the field ONLY if:<br>[rec_vaccine] = '1'	Get a vaccine (for flu, pneumonia, etc)	radio (Matrix), Required<br>1 Not at all likely1<br>2 2<br>3 3<br>4 4<br>5 5<br>6 6<br>7 Very likely7
159	six_mon_weight_loss<br>Show the field ONLY if:<br>[rec_weight] = '1'	Lose weight	radio (Matrix), Required<br>1 Not at all likely1<br>2 2<br>3 3<br>4 4<br>5 5<br>6 6<br>7 Very likely7
160	six_mon_blood_pressure<br>Show the field ONLY if:<br>[rec_bloodpressure] = '1'	Lower my blood pressure	radio (Matrix), Required<br>1 Not at all likely1<br>2 2<br>3 3

© 2021 Taksler GB et al. *JAMA Network Open.*

			4 4<br>5 5<br>6 6<br>7 Very likely7
161	six_mon_lower_sugar<br>Show the field ONLY if:<br>[rec_bloodsugar] = '1'	Lower my blood sugar	radio (Matrix), Required<br>1 Not at all likely1<br>2 2<br>3 3<br>4 4<br>5 5<br>6 6<br>7 Very likely7
162	six_mon_lower_cholesterol<br>Show the field ONLY if:<br>[rec_cholesterol] = '1'	Lower my cholesterol	radio (Matrix), Required<br>1 Not at all likely1<br>2 2<br>3 3<br>4 4<br>5 5<br>6 6<br>7 Very likely7
163	six_mon_aneurysm<br>Show the field ONLY if:<br>[rec_aneurysm] = '1'	Check for an aneurysm	radio (Matrix), Required<br>1 Not at all likely1<br>2 2<br>3 3<br>4 4<br>5 5<br>6 6<br>7 Very likely7
164	six_mon_breast<br>Show the field ONLY if:<br>[sex] = '2' and [rec_breastcancer] = '1'	Check for breast cancer(Get a mammogram)	radio (Matrix), Required<br>1 Not at all likely1<br>2 2

© 2021 Taksler GB et al. *JAMA Network Open.*

			3 3<br>4 4<br>5 5<br>6 6<br>7 Very likely7
165	six_mon_cervical<br>Show the field ONLY if:<br>[sex] = '2' and [rec_cervicalcancer] = '1'	Check for cervical cancer(Get a PAP test)	radio (Matrix), Required<br>1 Not at all likely1<br>2 2<br>3 3<br>4 4<br>5 5<br>6 6<br>7 Very likely7
166	six_mon_colerectal<br>Show the field ONLY if:<br>[rec_colorectal] = '1'	Check for colon cancer(Get a colonoscopy or atest for blood in your stool)	radio (Matrix), Required<br>1 Not at all likely1<br>2 2<br>3 3<br>4 4<br>5 5<br>6 6<br>7 Very likely7
167	six_mon_lung<br>Show the field ONLY if:<br>[rec_lung] = '1'	Check for lung cancer	radio (Matrix), Required<br>1 Not at all likely1<br>2 2<br>3 3<br>4 4<br>5 5<br>6 6<br>7 Very likely7
168	six_mon_aspirin<br>Show the field ONLY if:<br>[rec_aspirin] = '1'	Take aspirin	radio (Matrix), Required<br>1 Not at all likely1

© 2021 Taksler GB et al. *JAMA Network Open.*

			2 2<br>3 3<br>4 4<br>5 5<br>6 6<br>7 Very likely7
169	six_mon_std<br>Show the field ONLY if:<br>[rec_std] = '1'	Test for a sexually transmitted disease	radio (Matrix), Required<br>1 Not at all likely1<br>2 2<br>3 3<br>4 4<br>5 5<br>6 6<br>7 Very likely7
170	six_mon_smoking<br>Show the field ONLY if:<br>[rec_smoking] = '1'	Quit smoking	radio (Matrix), Required<br>1 Not at all likely1<br>2 2<br>3 3<br>4 4<br>5 5<br>6 6<br>7 Very likely7
171	thankyou5mins	Thank you!	descriptive
172	choice_options	Section Header: *Now, we would like to ask you how easy or difficult it is to make a choice about your preventive care. Considering the preventive care options you are interested in doing, please answer the following questions. You can use a scale from*	radio (Matrix), Required<br>1 Strongly Disagree1<br>2 Disagree2<br>3 Neither Agree Nor Disagree3<br>4 Agree4<br>5 Strongly Agree5

© 2021 Taksler GB et al. *JAMA Network Open.*

		"Strongly Disagree" to "Strongly Agree."<br>I know which preventive care options are available to me.	
173	choice_benefits	I know the benefits of each preventive care option.	radio (Matrix), Required<br>1 Strongly Disagree1<br>2 Disagree2<br>3 Neither Agree Nor Disagree3<br>4 Agree4<br>5 Strongly Agree5
174	choice_risk	I know the risks and side effects of each preventive care option.	radio (Matrix), Required<br>1 Strongly Disagree1<br>2 Disagree2<br>3 Neither Agree Nor Disagree3<br>4 Agree4<br>5 Strongly Agree5
175	choice_benefits_matter	I am clear about which benefits matter most to me.	radio (Matrix), Required<br>1 Strongly Disagree1<br>2 Disagree2<br>3 Neither Agree Nor Disagree3<br>4 Agree4<br>5 Strongly Agree5
176	choice_risks_matter	I am clear about which risks and side effects matter most to me.	radio (Matrix), Required<br>1 Strongly Disagree1<br>2 Disagree2<br>3 Neither Agree Nor Disagree3<br>4 Agree4<br>5 Strongly Agree5
177	choice_important	I am clear about which is more important to me(the benefits	radio (Matrix), Required<br>1 Strongly Disagree1<br>2 Disagree2

© 2021 Taksler GB et al. *JAMA Network Open.*

		or the risks and side effects) .	3 Neither Agree Nor Disagree3<br>4 Agree4<br>5 Strongly Agree5
178	choice_support	I have enough support from others to make a choice about my preventive care.	radio (Matrix), Required<br>1 Strongly Disagree1<br>2 Disagree2<br>3 Neither Agree Nor Disagree3<br>4 Agree4<br>5 Strongly Agree5
179	choice_pressure	I am choosing my preventive care without pressure from others.	radio (Matrix), Required<br>1 Strongly Disagree1<br>2 Disagree2<br>3 Neither Agree Nor Disagree3<br>4 Agree4<br>5 Strongly Agree5
180	choice_advice	I have enough advice to make a choice about my preventive care.	radio (Matrix), Required<br>1 Strongly Disagree1<br>2 Disagree2<br>3 Neither Agree Nor Disagree3<br>4 Agree4<br>5 Strongly Agree5
181	choice_best	I am clear about the best choice for me.	radio (Matrix), Required<br>1 Strongly Disagree1<br>2 Disagree2<br>3 Neither Agree Nor Disagree3<br>4 Agree4<br>5 Strongly Agree5
182	choice_choose	I feel sure about which preventive care options to choose.	radio (Matrix), Required<br>1 Strongly Disagree1<br>2 Disagree2<br>3 Neither Agree Nor Disagree3

© 2021 Taksler GB et al. *JAMA Network Open.*

			4 Agree4<br>5 Strongly Agree5
183	choice_easy	The decision about my preventive care is easy for me to make.	radio (Matrix), Required<br>1 Strongly Disagree1<br>2 Disagree2<br>3 Neither Agree Nor Disagree3<br>4 Agree4<br>5 Strongly Agree5
184	choice_informed	I feel I have made an informed choice about my preventive care.	radio (Matrix), Required<br>1 Strongly Disagree1<br>2 Disagree2<br>3 Neither Agree Nor Disagree3<br>4 Agree4<br>5 Strongly Agree5
185	choice_importance	My preventive care decision shows what is important to me.	radio (Matrix), Required<br>1 Strongly Disagree1<br>2 Disagree2<br>3 Neither Agree Nor Disagree3<br>4 Agree4<br>5 Strongly Agree5
186	choice_stick	I expect to stick with my preventive care decision.	radio (Matrix), Required<br>1 Strongly Disagree1<br>2 Disagree2<br>3 Neither Agree Nor Disagree3<br>4 Agree4<br>5 Strongly Agree5
187	choice_satisfied	I am satisfied with my preventive care decision.	radio (Matrix), Required<br>1 Strongly Disagree1<br>2 Disagree2<br>3 Neither Agree Nor Disagree3<br>4 Agree4

			<table><tr><td>5</td><td>Strongly Agree5</td></tr></table>
188	trust	Section Header: *Thank you! We are almost done.*<br><br>On a scale of 1-10, how much do you trust the doctor (or other health care professional) from today's visit?	radio (Matrix), Required<table><tr><td>1</td><td>No trust at all1</td></tr><tr><td>2</td><td>2</td></tr><tr><td>3</td><td>3</td></tr><tr><td>4</td><td>4</td></tr><tr><td>5</td><td>5</td></tr><tr><td>6</td><td>6</td></tr><tr><td>7</td><td>7</td></tr><tr><td>8</td><td>8</td></tr><tr><td>9</td><td>9</td></tr><tr><td>10</td><td>Complete trust10</td></tr></table>
189	known_doctor	How long have you known the doctor (or other health care professional) from today's visit?	radio, Required<table><tr><td>1</td><td>Less than a year</td></tr><tr><td>2</td><td>1 to 2 years</td></tr><tr><td>3</td><td>3 to 5 years</td></tr><tr><td>4</td><td>More than 5 years</td></tr></table>
190	frequency_of_visit	How often do you see the doctor (or other health care professional) from today's visit?	radio, Required<table><tr><td>1</td><td>This was my first time</td></tr><tr><td>2</td><td>Less than once a year</td></tr><tr><td>3</td><td>About once a year</td></tr><tr><td>4</td><td>About 2 times a year (every 6 months)</td></tr><tr><td>5</td><td>About 3-4 times a year (every 3 to 4 months)</td></tr><tr><td>6</td><td>More than 4 times a year (every 2 months or more often)</td></tr></table>
191	hispanic	Section Header: *These are the final questions.*<br><br>Are you Hispanic or Latino/a?	yesno, Required<table><tr><td>1</td><td>Yes</td></tr><tr><td>0</td><td>No</td></tr></table>
192	race	What is your race?	checkbox, Required<table><tr><td>1</td><td>race___1</td><td>White or Caucasian</td></tr></table>

© 2021 Taksler GB et al. *JAMA Network Open.*

		(Please select all that apply)	2 | race___2 | Black or African American<br>3 | race___3 | Native American, American Indian or Alaska Native<br>4 | race___4 | Pacific Islander or Native Hawaiian<br>5 | race___5 | Other:(please specify)
193	other_race	Other Race(please specify)	text
194	martial_status	What is your martial status?	radio, Required<br>1 | Married or living with a civil/domestic partner<br>2 | Widowed<br>3 | Divorced<br>4 | Separated from your spouse or partner<br>5 | Never married or in a civil/domestic partnership
195	education	What is the highest level of education you have completed?	radio, Required<br>1 | Less than high school<br>2 | High school(Diploma or GED)<br>3 | Some college<br>4 | College degree<br>5 | Master's degree<br>6 | Doctoral or Professional degree
196	health_beliefs	Please answer this last question about your health beliefs.<br><br>In your opinion, would you say that your health is:	radio, Required<br>1 | Excellent<br>2 | Very good<br>3 | Good<br>4 | Fair<br>5 | Poor
197	final_pre	Section Header:<br>We are now done with the survey.	descriptive

© 2021 Taksler GB et al. *JAMA Network Open.*

		In appreciation of your time today, we will mail you a $25 gift card.<br><br>In this survey, we asked you questions about things you might do to improve your health. Please understand that we were NOT making any suggestions for your health care. We were just gathering information for a research study.<br><br>If you would like to know more about anything you read in this survey, please talk to your doctor or other health care professional.<br><br>Thank you for your time.	
198	time_end	End time	text (datetime_seconds_ymd)<br>Custom alignment: LV<br>Field Annotation: @NOW @HIDDEN
199	patient_survey_complete	Section Header: *Form Status*<br><br>Complete?	dropdown<br>0 Incomplete<br>1 Unverified<br>2 Complete

© 2021 Taksler GB et al. *JAMA Network Open.*
