# Supplementary material for: Effect of Individualized Preventive Care Recommendations vs Usual Care on Patient Interest and Use of Recommendations: A Pilot Randomized Clinical Trial
Source: JAMA Netw Open. 2021 Nov 2;4(11):e2131455. doi: 10.1001/jamanetworkopen.2021.31455 (PMC8564576; doi:10.1001/jamanetworkopen.2021.31455)
Supplement: Supplement 3. — Data Sharing Statement [file jamanetwopen-e2131455-s003.pdf]

Taksler GB, Hu B, DeGrandis F, et al. Effect of individualized preventive care recommendations vs usual care on patient interest and use of recommendations. *JAMA Netw Open.* 2021;4(11):e2131455. doi:10.1001/jamanetworkopen.2021.31455

Data Sharing Statement

Data
Data available: No

Additional Information
Explanation for why data not available: Participants did not consent to data sharing
